# High-throughput cytological profiling uncovers genotype-phenotype associations in *Mycobacterium tuberculosis* clinical isolates

**DOI:** 10.1128/msystems.00972-25

**Published:** 2025-10-09

**Authors:** Qingyun Liu, Yue J. Liu, Ruiyuan Liu, Peter H. Culviner, Xin Wang, Ian D. Wolf, Ken Chen, Yiwang Chen, Yi Xiao, Guiming Zhang, Rongfeng Sun, Shoko Wakabayashi, Nicole C. Howard, Mingyu Gan, Eric J. Rubin, Sarah M. Fortune, Junhao Zhu

**Affiliations:** 1 Department of Genetics, University of North Carolina at Chapel Hill 2331, Chapel Hill, North Carolina, USA; 2 Department of Microbiology and Immunology, University of North Carolina at Chapel Hill 2331, Chapel Hill, North Carolina, USA; 3 Department of Immunology and Infectious Diseases, Harvard T.H. Chan School of Public Health, Boston, Massachusetts, USA; 4 Laboratory of Pathogen Microbiology and Immunology, Institute of Microbiology, Chinese Academy of Sciences, Beijing, China; 5 Key Laboratory of Medical Molecular Virology (MOE/NHC/CAMS), School of Basic Medical Sciences, Shanghai Medical College, Shanghai Institute of Infectious Disease and Biosecurity, Fudan University, Shanghai, China; University of Wisconsin-Madison, Madison, Wisconsin, USA; Southwest University, Beibei, Chongqing, China

**Keywords:** *Mycobacterium tuberculosis*, evolutionary biology, phenotypic variation, population genetics, fluorescent image analysis, Mycobacterium, high-throughput imaging

## Abstract

Understanding the functional impact of bacterial genetic diversity is crucial for linking pathogen variants to clinical outcomes. Here, we introduce a high-throughput cytological profiling pipeline optimized for *Mycobacterium tuberculosis* (Mtb) clinical strains, integrating OD-calibrated feature analysis and high-content microscopy. Our system quantifies single-bacterium morphological and physiological traits related to DNA replication, redox state, carbon metabolism, and cell envelope dynamics. Applied to 64 Mtb clinical isolates from lineages 1, 2, and 4, the approach revealed that cytological phenotypes recapitulate genetic relationships and exhibit both lineage- and density-dependent dynamics. Notably, we identified a link between a convergent “small cell” phenotype and a convergent *ino1* mutation that is associated with the presence of an antisense transcript, suggesting a potential non-canonical regulatory mechanism under selection. In summary, we present a resource-efficient approach for mapping Mtb’s phenotypic landscape, uncovering cellular traits that underlie its evolution and providing new insights into the functional consequences of bacterial genetic diversity.

**IMPORTANCE:**

Understanding how genetic variation in *Mycobacterium tuberculosis* (Mtb) shapes its physical traits is essential to unraveling the evolution of this global pathogen. Here, we introduce a systematically optimized, high-throughput imaging platform for the comprehensive characterization of Mtb clinical strains. We demonstrate that Mtb’s phenotypic manifestation is shaped by both genetic background and culture density. By accounting for these factors, our analysis linked distinct cellular dynamics to specific lineages, sublineages, and even single nucleotide variations. Notably, we linked a recurring mutation to a unique cell-shortening phenotype, finding that it potentially acts by creating a cryptic antisense transcript. This platform provides a powerful framework for systematically dissecting the physiological dynamics underlying Mtb evolution and identifying new therapeutic vulnerabilities of this deadly pathogen.

## INTRODUCTION

The recent expansion of whole-genome sequencing for bacterial pathogens has revealed extensive genetic diversity and its potential impact on critical clinical outcomes, including host infection, treatment efficacy, and transmission (1–3). However, despite the abundance of genomic data, phenotypic investigations often lag behind, leaving much of the phenotypic landscape unexplored and the functional impact of most genetic variations unknown (4, 5). This paucity of phenotypic data—and more importantly, the lack of methods to interrogate the phenotypic landscape of clinical isolates at scale—has limited our ability to assess the impact of bacterial genetic diversity.

One example of the imbalance between genomic and phenotypic data is the respiratory pathogen *Mycobacterium tuberculosis* (Mtb), which causes active tuberculosis in over 10 million people and is responsible for more than a million deaths annually (6). A decade of global TB genetics and epidemiology research has generated over 100,000 whole-genome sequencing data of Mtb clinical isolates (7), which empowered subsequent genome-wide association studies (GWAS) to draw critical connections between specific genetic variations and clinical observations, such as drug resistance (8–10), *in vivo* fitness (11), and transmissibility (12). Nevertheless, these associations account for only a small fraction of the genomic variations observed in the Mtb population and are often limited by the effect size of individual variants. However, recent studies have shown that mutations in different genes within the same pathway can result in similar phenotypes (10, 13), suggesting that direct phenotyping of clinical strains may provide a complementary strategy to uncover the novel, translationally relevant biology that bridges bacterial genotypes with phenotypes.

Existing methods, such as transposon sequencing (Tn-Seq) (14), CRISPR-interference sequencing (15), and genetic barcode tracing (16), are powerful in defining the fitness landscape across a wide array of genetic variants or clinical isolates. However, these methods predominantly capture growth fitness as bulk outcomes, obscuring the underlying physiological changes at the cellular level—which are especially interesting given that mycobacteria can exhibit single-cell heterogeneity that contributes to survival under stress conditions (17, 18). Microscopy remains one of the most powerful tools for characterizing bacterial phenotypes at a single-cell level. Recent advances in microscope automation have enabled scalable imaging platforms to interrogate antibiotic susceptibility, cell morphology, and other traits across diverse bacterial species (19–22). These methods have been proven useful in identifying features that can differentiate samples associated with diverse genetic backgrounds or subjected to varying conditions (19, 23, 24). Nevertheless, challenges remain in Mtb cytological profiling, including how to effectively probe a BSL-3 pathogen prone to form large cell aggregates (21, 25, 26) and how to maximize the quantity and the diversity of cellular traits pertaining to Mtb physiology. Therefore, while various bacterial cytological profiling (BCP) approaches exist, none have yet been sufficiently optimized for Mtb research, underscoring the need for a tailored platform that can reliably capture the complex cellular heterogeneity and evolutionary dynamics of this critical human pathogen.

In this work, we described a high-throughput cytological profiling system systematically optimized for Mtb that readily captures comprehensive cytological profiles of 1,000–10,000 bacilli for up to 32 samples per hour. We tested this system using 64 Mtb clinical isolates and assessed feature variations within and between Mtb strains, which revealed substantial phenotypic diversity within and between strains. We further identified growth phase (culture density) as a variable affecting these physiological traits and developed a LOWESS-trendline-based interpolation model for feature normalization and cross-comparison of strains with distinct growth dynamics. We demonstrated that our system can discern lineage-, sublineage-, and even strain-specific phenotypic traits and associate a convergent phenotype to a certain genetic mutation. We provide this method as an open-access resource for microbiology laboratories.

## RESULTS

### Development and optimization of a high-throughput bacterial cytological profiling platform for Mtb

**Fig 1:**
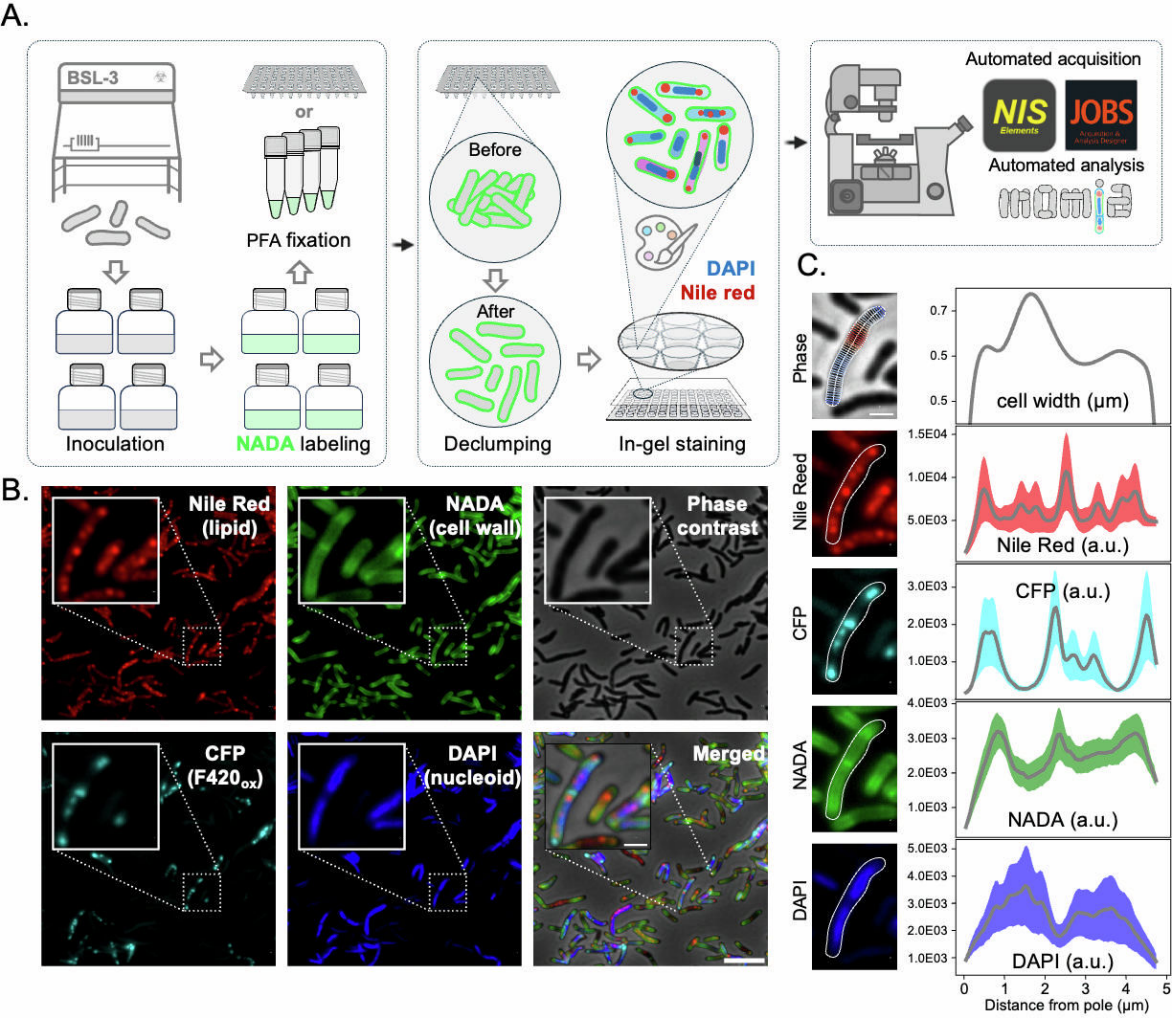
Overview of the high-throughput cytological profiling pipeline for Mtb clinical isolates. (**A**) Schematic representation of the experimental workflow. Mtb cultures were grown in BSL-3 lab and labeled with NADA prior to fixation with paraformaldehyde (PFA). Fixed samples underwent a de-clumping step to reduce bacterial aggregation before being subjected to in-gel staining with DAPI (DNA stain) and Nile Red (lipid stain). The prepared samples were imaged using an automated fluorescence microscopy system equipped with NIS Elements and JOBS software for high-throughput image acquisition and analysis. (**B**) Representative fluorescence microscopy images of Mtb cells stained with Nile Red (red, lipid staining), NADA (green, cell wall labeling), CFP/F420 autofluorescence (cyan, redox state indicator), and DAPI (blue, DNA staining). The phase contrast image (bottom right) shows the overlay of all fluorescence channels, highlighting the subcellular localization of various signals. Insets depict zoomed-in regions, illustrating single-cell level staining patterns. Scale bars: 5 and 1 microns for original and zoom-in views, respectively. (**C**) Subcellular profiling of individual Mtb cells. The left column displays five-channel images of a representative cell, highlighted in Fig. 1B. The top phase-contrast image is overlaid with its centerline and an evenly spaced orthogonal mesh used for quantification. The orthogonal mesh is pseudo-colored according to the measured width, with warmer colors indicating wider regions. The subsequent fluorescence images are overlaid with a subpixel outline (white lines). The right column presents corresponding quantitative profiles plotted against the distance from one cell pole. The cell width profile is defined by the length of the orthogonal mesh lines. For fluorescence signals, the solid gray line represents the mean intensity along the medial axis, while the shaded colored area indicates the standard deviation across the cell’s width at each point. Scale bar: 1 µm.

Existing bacterial cytological profiling platforms had been extensively tested against *E. coli*, *A. baumannii*, *S. aureus*, and more recently against Mtb (19, 21, 26). However, in our own, earlier attempts to establish a cytological profiling system for Mtb, we identified several technical limitations in adapting existing platforms to Mtb. First, high-throughput cytological profiling of bacterial cells requires immobilizing small bacilli for a prolonged amount of time to minimize focus drift and planar distortion. While multi-well imaging containers with functionalized (e.g., poly-L-lysine-coating) glass surfaces are broadly used for high-throughput imaging of eukaryotic cells, their utility for Mtb is constrained by the pathogen’s lipid-rich cell envelope, which results in highly variable adherence. To address this, we developed a customized 96-well molding tool set analogous to several published designs (27–29) and optimized its fabrication process for better cost-effectiveness and consistency (Fig. 1A; Fig. S1). The core components include two plastic plates with arrayed 96 wells, which can either be fabricated using commercial-grade 3D printers or directly purchased as part of the recyclable accessories of pipette tip boxes from VWR (Fig. S1A through E; Movie S1). Upon assembly, our molding system casts 96 arrayed pedestals, minimizing the risk of cross-well contamination (Fig. S1G and H). The assembled multi-well imaging plate is compatible with most motorized digital microscopes. Here, we adopted a previously established imaging platform enabled by a motorized Nikon Ti-E inverted microscope with minor optimizations to accommodate additional fluorescence channels and more fields of view (FOV) (25). The acquisition time of our standard imaging configuration (five channels for 24 FOVs) ranges from 3 to 3.5 minutes per sample, which sums up to about five hours for a full 96-well plate (Fig. 1).

The second challenge imposed by Mtb’s lipid-laden cell envelope is propensity to form large aggregates, which can skew image segmentation and population-level analyses. To mitigate clumping, we developed a xylene–Triton X-100 emulsion that effectively disperses Mtb clumps and concurrently quenches excess formaldehyde in fixed samples (Fig. S2A). This de-clumping method faithfully preserves the morphological and chemical fluorescence staining properties of mycobacterial cells (Fig. S2B and C), achieves uniform sampling of heterogeneously clumping samples, is reasonably time efficient (takes about 20 minutes to process a full 96-well plate), and requires only a multi-well-plate-compatible benchtop centrifuge.

Thirdly, we sought to optimize the fluorescence labeling strategy for Mtb samples. Many bio-orthogonal chemical fluorescence labeling tools had been developed for Mtb detection (30–32). Taking dye cost, signal intensity, spectrum compatibility, and staining consistency into consideration, we devised a panel of cost-efficient staining protocols for diverse cytological profiling applications. In general, these protocols consist of two staining stages: a pre-fixation cell wall labeling using fluorescent D-amino acids (FDAAs), and a post-fixation on-gel staining using target-specific, turn-on probes, such as DAPI and Nile Red. Compared to the conventional stain-wash-image routine, we chose on-gel staining because it is superior in maintaining staining signal after extended incubation. In addition to chemical fluorescence staining, we also leveraged the autofluorescence signals emitted by oxidized cytochrome F420, which is abundant in Mtb cells and associated with the redox homeostasis (26, 32).

Despite the overall effectiveness of our chemical declumping method, small chunks (about 2–10 cells) of bacteria aggregates were still prevalent in many samples. In many cases, cells within these small aggregates were irregularly stacked on one another, and there was insufficient contrast to facilitate accurate 2D segmentation. To minimize segmentation errors due to unresolved aggregates, uneven illumination, focus drift, and other rare forms of imaging anomalies, we rebuilt our published microscopy image analysis program, MOMIA (Mycobacteria Optimized Microscopy Image Analysis) (25), and implemented a trainable classifier to guide automated anomaly detection and removal (Fig. S3). This restructured Python package, now named MOMIA2, achieves efficient and accurate image segmentation, anomaly detection, and single-cell identification (Fig. 1A; Fig. S3). By default, MOMIA2 renders a diverse panel of quantitative features for each bacterial cell (Fig. S4), encompassing various categories, including (i) morphological features, such as cell size, length, and width; (ii) primary intensity features representing lipid droplet content, DNA content and stainability, and NADA staining intensity (pertaining to cell wall biosynthesis); and (iii) subcellular profiles, such as DNA morphology, lipid droplet distribution, and FDAA staining pattern along the cell axis. These single-bacterium metrics would enable us to not only query the populational statistics for each sample or isolate, but also to delve into the within-population heterogeneity of Mtb clinical strains.

### Cytological profiling of 64 clinical Mtb strains

**Fig 2:**
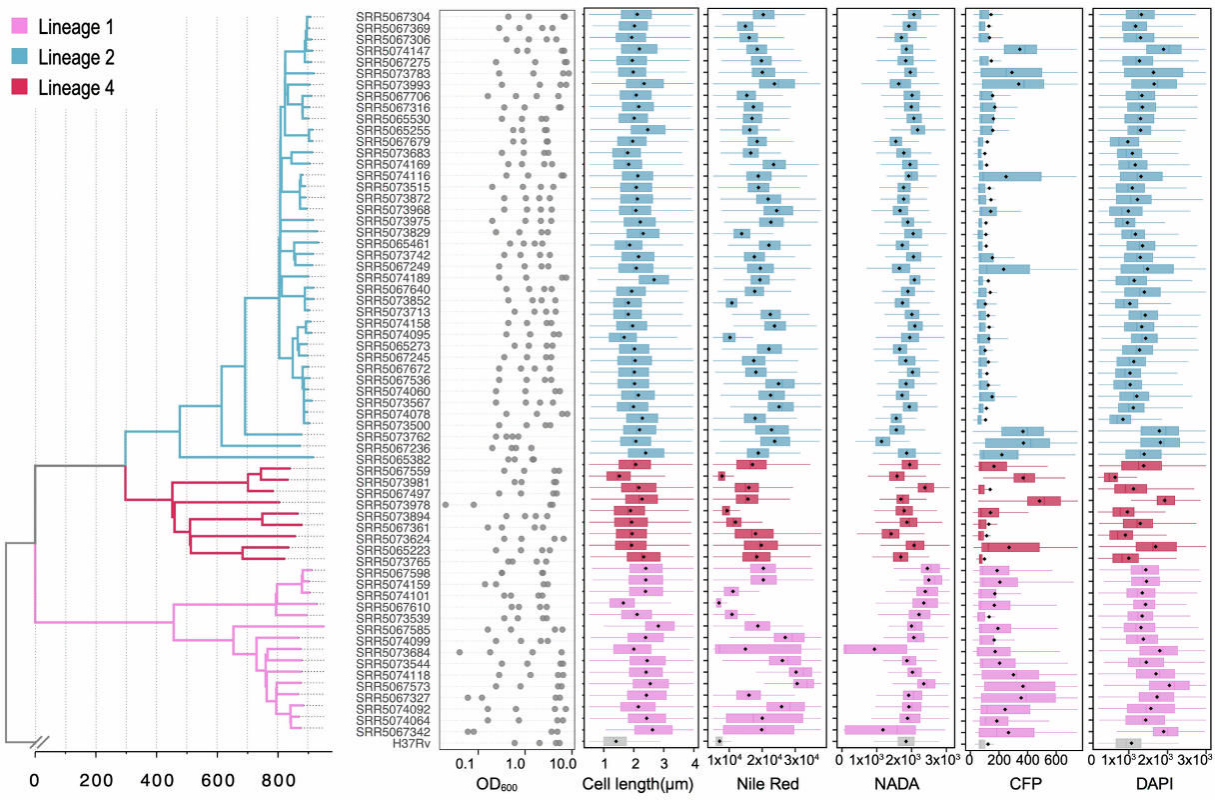
Phylogenetic structure and cytological feature variation across 64 Mtb isolates. (Left panel) Maximum-likelihood phylogenetic tree of 64 Mtb isolates, with branches colored by lineage: lineage 1 (pink), lineage 2 (blue), and lineage 4 (red). Isolate identifiers are listed next to the corresponding tips of the tree. (Middle panel) Scatter plot showing the OD_600_ values at which each isolate was sampled, capturing variation in growth phase across strains. (Right panels) Boxplots displaying the distribution of five key cytological features across isolates (harvested at day 6): cell length (µm), Nile Red fluorescence (lipid accumulation), NADA fluorescence (cell wall labeling), CFP fluorescence (redox state), and DAPI fluorescence (DNA staining). Each boxplot represents data from individual strains, with lineage-specific color coding matching the phylogenetic tree. Black dots indicate median values.

We hypothesized that systems phenotyping—such as bacterial cytological profiling (BCP)—of genetically diverse clinical isolates could unveil structured associations between the phenotypic landscape of these isolates and their genetic background. To test this, we selected 64 Mtb strains—including 63 from a previous collection in Ho Chi Minh City (33) and a lab reference strain H37Rv—for BCP analysis. These 64 strains comprise 15 lineage 1 strains, 40 lineage 2 strains, and 9 lineage 4 strains (Fig. 2), and were selected to represent genetic diversity at the levels of lineages, sublineages, and strains (Fig. 2). All 64 Mtb strains were cultured in standard 7H9 medium supplemented with 10% OADC, and bacterial samples were harvested during either the log phase or stationary phase. NADA, a fluorescent D-amino acid that incorporates into peptidoglycan layer of the cell wall, was added to the Mtb cultures for cell wall labeling. The Mtb strains were inoculated in fresh culture at an OD_600_ of 0.01, and the bacterial culture was harvested at three to four different time points to reflect the different growth phases (i.e., 5, 6, 12, and 15 days after inoculation). Because these clinical strains have different growth rates (Fig. S5), the OD_600_ states at harvest varied drastically across strains despite a similar starting inoculum. We successfully obtained an average of ~2,000 analyzable cells from each strain at each time point (566–15,091, median: 2,039). The imaging data were automatically segmented and analyzed using the MOMIA2 pipeline described above to obtain numeric features for each trait (Materials and Methods).

### Modeling growth-dependent dynamics of physiological features

**Fig 3:**
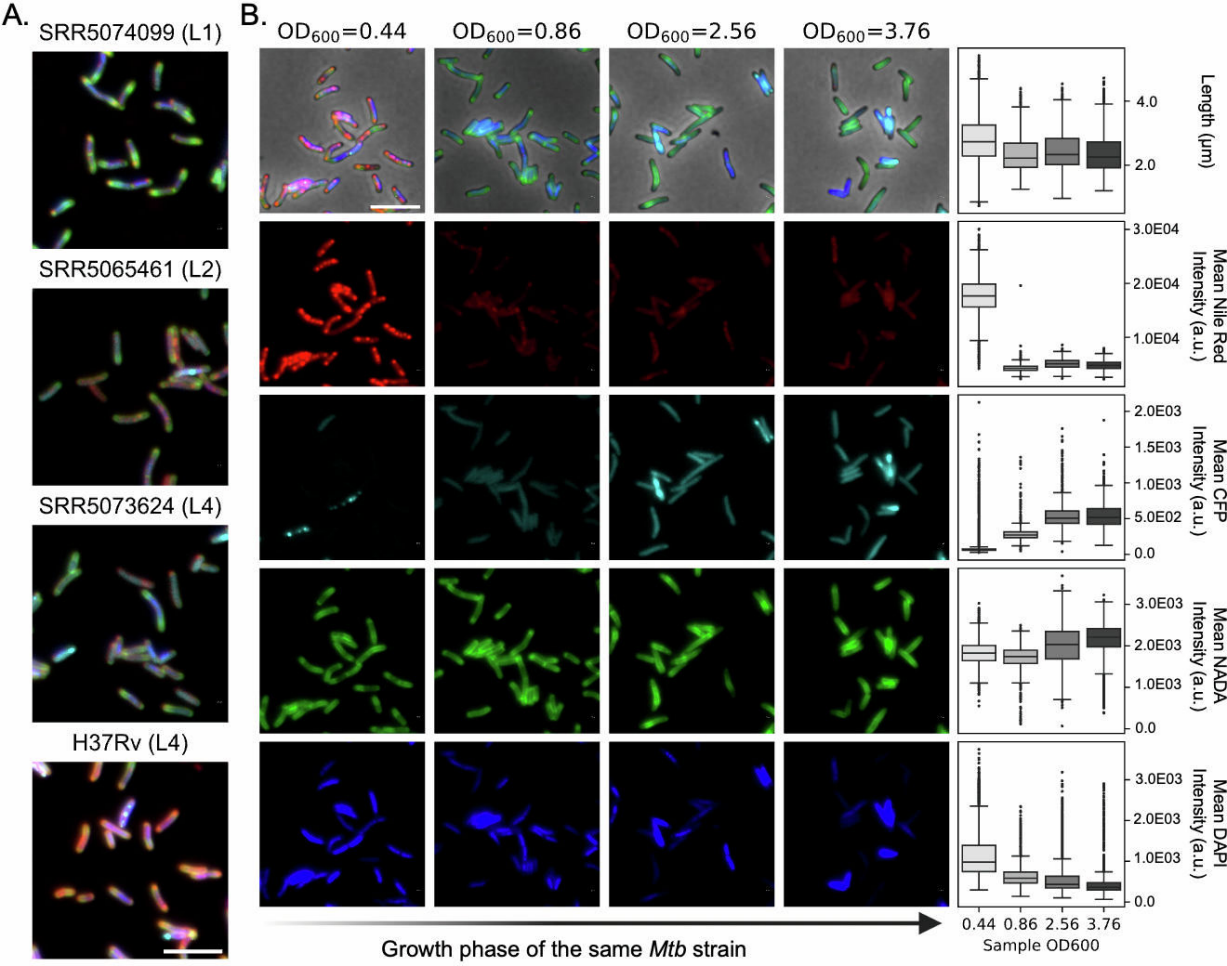
Cytological heterogeneity across Mtb strains and growth phases. (**A**) Representative fluorescence microscopy images of four Mtb strains from different lineages: L1 (SRR5074099), L2 (SRR5065461), L4 (SRR5073624), and the laboratory reference strain H37Rv (L4). Cells are stained with Nile Red (lipid inclusions, red), NADA (cell wall labeling, green), DAPI (DNA, blue), and CFP autofluorescence (redox state, cyan). Scale bar: 5 µm. (**B**) Growth phase-dependent cytological changes in an Mtb strain (SRR5074169) sampled at increasing OD_600_ values (0.44, 0.86, 2.56, and 3.76). The top row shows composite fluorescence and phase contrast images at each growth phase. Rows below show individual fluorescence channels for Nile Red, CFP (autofluorescence, NADA, and DAPI staining). Corresponding boxplots (right column) display the populational profiles of cell length and average fluorescence intensities of Nile Red staining, CFP autofluorescence, NADA incorporation, and DAPI staining across different growth phases. Scale bar: 5 µm.

**Fig 4:**
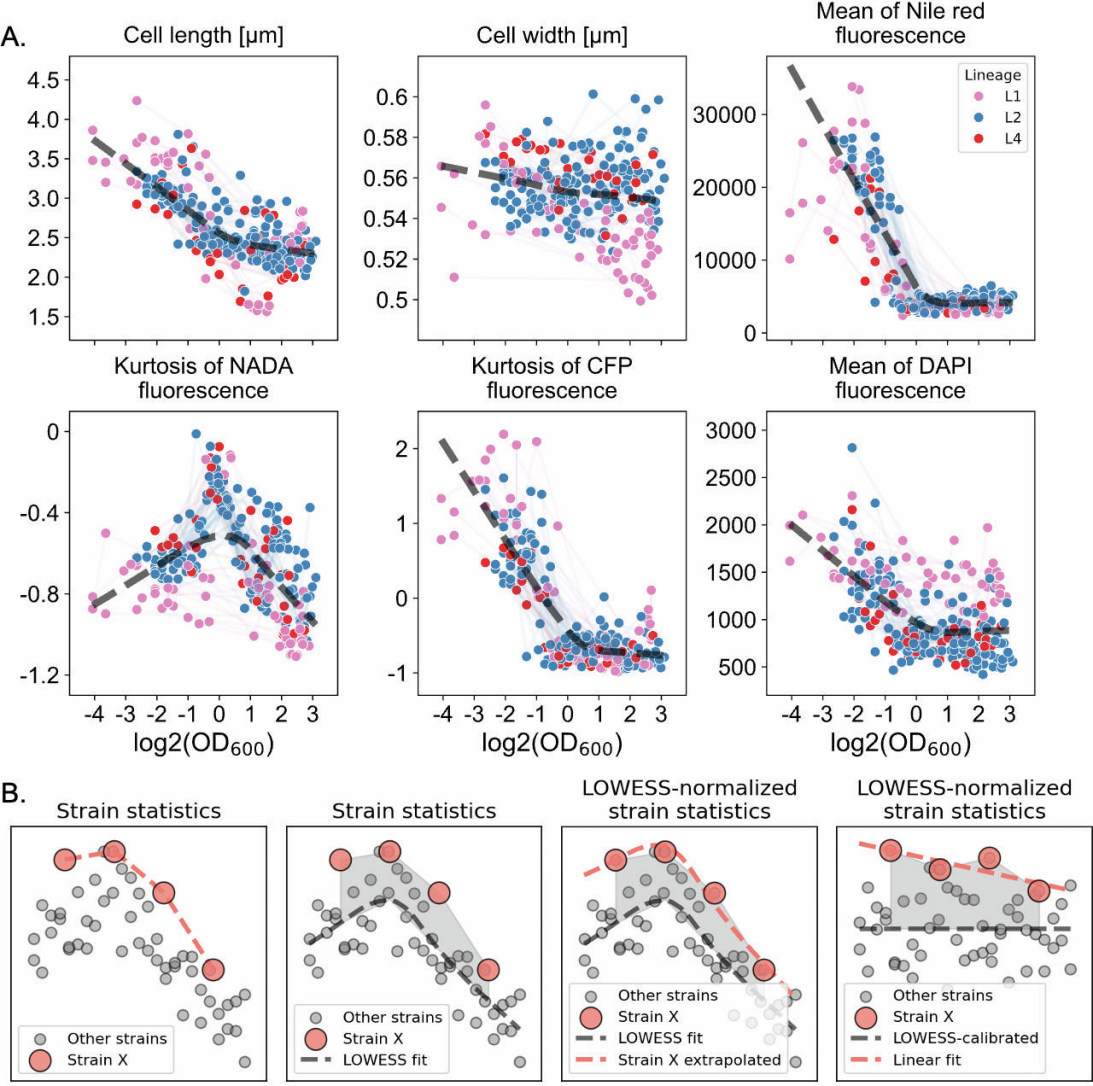
Growth phase-dependent dynamics of cytological features across Mtb lineages. (**A**) Scatter plots showing the relationship between OD_600_ (log2 scale) and key single-cell morphological and physiological features across Mtb isolates from lineage 1 (pink), lineage 2 (blue), and lineage 4 (red). Each point represents a strain-specific measurement. Features analyzed include cell length, cell width, Nile Red fluorescence (lipid content), NADA fluorescence kurtosis (cell wall remodeling), CFP fluorescence kurtosis (redox state variability), and DAPI fluorescence intensity (DNA content). Dashed gray lines indicate LOWESS smoothing to illustrate global trends. (**B**) A schematic illustrating the trendline-based normalization process. The relationship between log2-transformed OD_600_ and a given feature was modeled by LOWESS (black segmented line) to establish a baseline. This baseline was then used to compute the deviation of strain-level statistics from the global trend. The trendline-normalized strain-level statistics were subsequently fitted to a linear function, allowing for the interpolation and extrapolation of the relative feature profile at any given optical density.

At first glance, we observed extensive heterogeneity in bacterial features both across and within Mtb strains when aggregating data by strain. For example, the median cell length and width varied across stains and ranged from 1.55 to 4.25 microns and from 0.5 to 0.6 µm, respectively (Fig. 2). Fluorescence intensities reflecting NADA incorporation, DAPI staining, and F420 autofluorescence (CFP) showed substantial variation within strains, with average coefficients of variation (CV) of 22%, 54%, and 64%. These within-strain CVs were comparable to—or in some cases exceeded—the corresponding CVs observed across strains (23%, 40%, and 39%, respectively). The visually and statistically pronounced cytological variability among strains and the three lineages unveiled a substantial, yet previously uncharacterized, phenotypic diversification underlying Mtb clinical evolution (Fig. 2 and 3A). Nevertheless, as these strains were sampled at multiple growth stages, the bulk analysis depicted in Fig. 2 also reflects growth stage- and culture density–associated cytological variations, the principles of which remain largely undefined. Indeed, when examined at a single-strain level, we observed profound changes in cell morphology, lipid droplet content, F420 signal, NADA incorporation patterns, and DAPI staining intensities as culture density increased (Fig. 3B). To effectively compare the density-associated feature dynamics across strains, we calculated a set of populational statistics (median, coefficient of variation, and skewness) for each feature from all single cells within a sample and compared these metrics across different growth phases for all strain against OD_600_ values (Fig. 4A; Table S1). We observed significant correlations between bacterial features and OD_600_ values: cell length and DAPI signal appear to change monotonically with OD_600_, whereas the dynamics of the Nile Red signal (lipid droplet) demonstrated an “L” shape as it decreases rapidly after OD_600_ of 0.1 and reaches a steady state after OD_600_ ~1.0 (Fig. 4A). Interestingly, the kurtosis of cellular F420 autofluorescence (CFP), a statistical measure of the degree of fluorescence aggregation, also decreases as culture density increases, implying a dynamic subcellular reorganization of F420 pool in response to metabolic adaptation (Fig. 3B and 4A). These patterns were consistent across isolates, suggesting that although bacterial characteristics vary across growth phases, the underlying biological mechanisms driving these changes appear to be conserved among strains from genetically diverse lineages (34).

While it is not feasible to collect samples from all strains at exactly the same OD_600_ values, the feature values collected at different OD_600_ states can be leveraged to model the trajectory of phenotypic changes, given that the feature–OD_600_ dynamics appear to follow a conserved trend. Therefore, we posited that the samples collected from 4 OD_600_ points could be used to model the dynamic of physiological features of each strain. We employed a locally weighted scatterplot smoothing (LOWESS) method to model this trajectory, as it effectively captures nonlinear trends and smooths variability in the data (Fig. 4B) (Materials and Methods). We modeled the relationship between each physiological feature and culture density (OD_600_) using LOWESS smoothing and generated an interpolated trendline. This trendline was then used to normalize feature values across different growth phases, allowing us to estimate feature values at specific OD_600_ states for direct comparison across strains. This approach enabled us to model the feature–OD_600_ dynamics and their deviations from the global trend for each strain using linear functions (Materials and Methods; Table S2). By leveraging the calibrated feature–OD_600_ relationships, we extracted feature values at defined growth stages, including early log phase (OD_600_: 0.2), late log phase (OD_600_: 0.8), early stationary phase (OD_600_: 1.2), and late stationary phase (OD_600_: 2.0), enabling direct comparisons across strains (Materials and Methods).

### Physiological features can reflect differences specific to genetic backgrounds

We then implemented a feature selection analysis and identified 25 phenotypic traits that exhibited association with their lineage background (Fig. 4A; Fig. S6 and Table S3). We generated a two-dimensional UMAP embedding of the 25 phenotypic traits (Materials and Methods) and computed the pairwise UMAP distance between isolates. We observed a significant positive correlation between pairwise genetic distance and phenotypic distance (Fig. 4B). There were some features that had patterns recapitulating strain lineage (Fig. 4A). Among those features, we found that the coefficient of variation (CV) of DAPI staining intensity made the highest contributions to lineage separation (Fig. 4C; Fig. S6D). Upon closer inspection, we found that strains from the three lineages exhibited comparable DNA staining profiles at optical densities below 0.1 (Fig. 3A; Fig. S7A). However, as L2 and L4 strains transitioned from the early exponential growth phase to the stationary phase, their DAPI staining intensities dropped dramatically along with a substantially increased variability (CV) in single-cell DAPI staining profiles (Fig. 4D; Fig. S7A and B). In contrast, L1 isolates manifested much milder changes in their DNA stainability as culture density increases (Fig. 4D; Fig. S7A and B). Notably, the altered DAPI staining profiles of L2 and L4 isolates were largely due to the emergence and expansion of DAPI-low subpopulations as culture density increased, implying potential phenotypic bifurcation within the culture (Fig. 4D; Fig. S7C). Previous works on H37Rv (L4 background) also revealed differential stainability of a homogeneous Mtb culture, albeit with different dyes (21, 35). Since the cells were killed and fixed with paraformaldehyde and examined by microscopy shortly after fixation, the lineage-specific differences in DNA stainability by DAPI are unlikely to result from DNA degradation. Instead, these differences may reflect intrinsic physiochemical properties of the three Mtb lineages. Specifically, a subpopulation of L2 and L4 strains—but not L1—may undergo cell envelope remodeling during stationary phase adaptation, leading to a reduced permeability to the hydrophilic DAPI molecule. Apart from differences in DAPI uptake, we also observed that L1 cells are slightly “slimmer” than those from the other two lineages. While both phenotypes support the hypothesis that L1 Mtb strains may differ from L2 and L4 in cell wall architecture and composition, further investigation is required to elucidate the underlying mechanisms.

**Fig 5:**
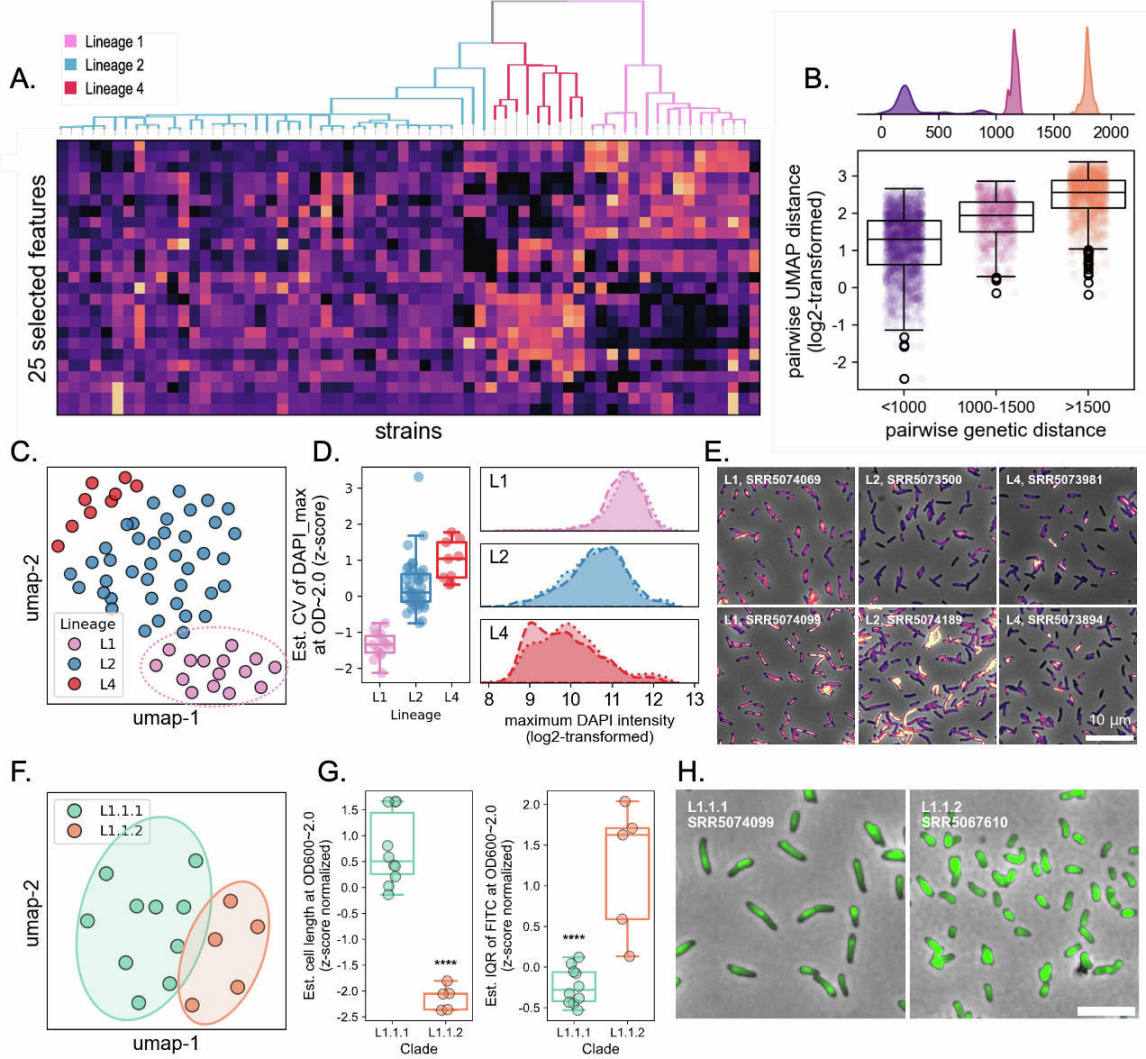
Phylogenetic structuring of cytological features in Mtb clinical isolates. (**A**) Heatmap of 25 selected cytological features across Mtb strains. (**B**) Relationship between genetic and phenotypic distances. (Top) Density plots of pairwise genetic distances between strains. (Bottom) Boxplot showing log2-transformed UMAP distances between strains categorized by genetic distance bins, indicating that phenotypic similarity corresponds to genetic relatedness. (**C**) UMAP projection of Mtb strains based on cytological features, showing lineage-specific clustering. Lineage 1.1.2 strains (dotted oval) form a distinct subcluster. (**D**) Differences in DAPI fluorescence intensity across lineages. (Left) Boxplot showing the coefficient of variation (CV) in maximum DAPI intensity. (Right) Density plots of maximum DAPI fluorescence (log2-transformed), indicating increased variability in lineage 2 and 4 strains. (**E**) Representative fluorescence microscopy images of Mtb strains from different lineages, illustrating lineage-specific differences in cellular morphology and staining patterns. Cells are stained with Nile Red (lipid inclusions, red), NADA (cell wall, green), and DAPI (DNA, blue). Scale bar: 10 µm. (**F**) UMAP projection highlighting subclade separation within lineage 1, distinguishing L1.1.1 and L1.1.2 strains. (**G**) Boxplots comparing extrapolated cell length (left) and FITC signal variability (right) between L1.1.1 and L1.1.2 strains. (**H**) Representative microscopy images of L1.1.1 (SRR5074099) and L1.1.2 (SRR5067610) strains, showing morphological differences and distinct NADA staining (pseudo-colored green) patterns. Scale bar: 10 µm.

In addition to the observed phenotypic segregation by their lineage background, we also observed phenotypic subclusters within each lineage. This suggests that clade- or even strain-specific mutations have caused deviations from their typical lineage behaviors. For example, we found that L1.1.1 and L1.1.2 were well separated in UMAP space (Fig. 5F through H). After analyzing the full set of OD-calibrated features, we identified a distinct trait that differentiates these two sublineages: L1.1.2 strains are significantly shorter than L1.1.1 strains upon entering the stationary phase (OD_600_ > 1.0). L1.1.2 strains are also more heterogeneous in terms of NADA staining (Fig. 5G and H), indicating that the bacterial cells of L1.1.2 strains differentiate into subpopulations that were differentially active in NADA incorporation.

### A convergent “small cell” phenotype links to a homoplastic mutation

**Fig 6:**
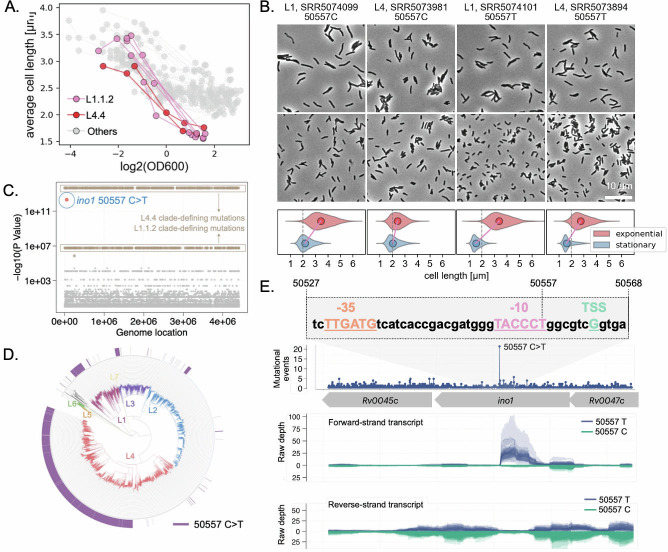
A convergent “small-cell” phenotype is associated with a recurrent mutation in *ino1* linked to antisense transcription. (**A**) Growth-dependent changes in average cell length across 64 Mtb strains. Seven strains display a marked cell length collapse in stationary phase, highlighted in pink (L1.1.2) and red (L4.4). (**B**) Representative phase-contrast images (top) and cell length distributions (bottom) for four strains with the *ino1* 50557C>T mutation. Violin plots compare cell lengths in exponential (blue) vs. stationary (red) phase. (**C**) Manhattan plot showing GWAS results for stationary-phase cell length. The ino1 50557C>T mutation is the top hit (*P* = 4.76 × 10⁻¹³). Clade-defining mutations are indicated in brown. (**D**) Phylogenetic distribution of the *ino1* 50557C>T mutation across a global Mtb collection. (**E**) Top: DNA sequence context around position 50557 suggests the C>T mutation introduces a new −10 promoter motif (TACCT) adjacent to a preexisting −35 motif (TTGATG). Bottom: RNA-Seq data from ~200 clinical isolates show antisense transcript coverage at the *ino1* locus only in strains carrying the 50557C>T mutation, suggesting mutation-associated antisense expression. Forward and reverse strand coverages are shown separately.

While we recognized that *Mtb* cells generally shorten when transitioning to stationary phase—a phenomenon termed reductive cell division (Fig. 4A)—the extreme cell size reduction in L1.1.2 strains stood out from the expected distribution of other Mtb isolates (Fig. 6A). In addition to L1.1.2, we identified two L4 strains exhibiting similar stationary phase-associated cell shortening (Fig. 6A and B), suggesting a convergent phenotype arising in distinct genetic backgrounds. Compared to other Mtb strains, which maintain an average stationary-phase cell length of ~2.4 µm, these seven “small cell” isolates shrank to ~1.6 µm (*P* = 0.033 by Mann–Whitney *U* test).

We hypothesized that this “small cell” phenotype is genetically determined and conducted a gene-level GWAS analysis to identify the potential genetic basis. Interestingly, all seven “small cell” strains shared the same *ino1* mutation (50557C>T) (*P* = 4.76E−13) (Fig. 6C), resulting in a Gly-to-Arg substitution at codon 190. *ino1* encodes inositol-1-phosphate synthase, an essential enzyme in *Mtb* involved in signal transduction pathways that regulate cell growth (36, 37). Given that homoplastic mutations in *Mtb* are rare (~1% of all variable sites) and typically occur in genes under positive selection (38), we reasoned that *ino1* might be a target of natural selection. However, dN/dS analysis of *ino1* across the full gene body indicated purifying selection (dN/dS = 0.53). This suggests that *ino1* itself may not be under direct adaptive evolution. To determine whether selection was acting on a specific site rather than the entire gene, we examined homoplastic mutations in this locus across a collection of ~50,000 *Mtb* isolates (10). Notably, the *ino1* 50557C>T mutation occurred at least 21 times independently, a level of convergence that sharply contrasted with other sites in the same gene (Fig. 6D and E). This frequent recurrence suggests that natural selection may be acting specifically on this site, potentially conferring a non-canonical function.

At the protein level, the 50557C>T mutation converts the Gly190 codon (GGG) to an arginine codon (AGG), which is rarely used across *Mtb* coding genome (encode about 4% of all arginines), while the alternative mutation at the same position (50557C>G), which would create a much more common arginine codon (CGG, encoding about 34% of all arginines), is not found across our global *Mtb* collection. The exclusive presence of the variant that generates a translationally suboptimal codon in clinical isolates suggests that the primary selective pressure acts on the non-coding function of this nucleotide, rather than on the G190R amino acid change in the Ino1 protein. Upon inspecting the surrounding DNA sequence, we found that the 50557C>T mutation introduces a new “TANNNT” motif (TACCCT), which could serve as a −10 promoter element by pairing with an existing −35 motif (“TTGATG”) (Fig. 6E). This suggests that the mutation may generate an antisense transcript overlapping *ino1*. Analysis of RNA-Seq data from ~200 clinical *Mtb* strains, including 20 strains from our phenotypic data set, confirmed that this mutation was exclusively associated with antisense RNA expression, which was absent in wild-type strains (Fig. 6E). Promoter reporter assays in a non-pathogenic surrogate, *M. smegmatis*, further confirmed that only the 50557T variant, but not the other three nucleotides, creates a functional promoter that drives strong downstream expression, consistent with the formation of a TACCCT −10 consensus sequence (Fig. S8). Thus, our high-throughput phenotyping has identified a convergent cellular phenotype associated with a recurrent regulatory mutation that generates an antisense RNA. Further investigation is needed to elucidate the function of this antisense RNA and its role in natural selection.

## DISCUSSION

In this work, we designed an agarose molding 96-well plate system for automatic profiling of features of bacterial cells in an automated manner. This system is cost-efficient compared to commercial multi-well glass-bottom imaging plates and is generally easy to work with. While this system is analogous to molding scaffolds reported in previous works, there are two noteworthy technical differences/improvements. First, instead of using plain PBS-buffered agarose to cast multi-well agarose pads to immobilize the cells, we added DAPI and Nile Red dyes directly to the melted agarose before gelation. Compared to conventional methods, which require the staining of cells before seeding, our in-gel staining method demands fewer pipetting steps and maintains dye staining equilibrium at saturating concentrations. Second, we implemented an emulsion-based cell dissociation method to combat frequent mycobacteria cellular aggregation and to increase cell accessibility. Together, these technical optimizations enabled us to acquire dozens of cellular features from hundreds of Mtb cells in a single multi-channel microscopy snapshot.

The feature data from multiple growth phases of Mtb strains indicated that bacterial characteristics, including cell length, width, and other physiological traits, are not fixed parameters for a given strain. As culture density increases, shifts in nutrient availability, waste accumulation, and bacterial metabolic activity occur in a tightly regulated fashion, presumably driving corresponding changes in cellular features. In this study, we leveraged sparse temporal sampling to capture culture density-associated cytological variations across different Mtb strains. Our analyses highlight that culture density is a key determinant of Mtb’s cytological feature dynamics and that the substantial differences in density-dependent feature profiles among isolates reflect their underlying genetic backgrounds. Nile Red staining exhibited the most striking density-associated feature dynamics. As culture density increased, intracellular Nile Red fluorescence—likely derived from neutral lipids stored in lipid droplets—initially surged before gradually declining back to its baseline. This pattern is reminiscent of a recent preprint from the Stanley group (39), which proposed that mycobacteria actively form lipid droplets upon entering lipid-rich fresh media and subsequently utilize these intracellular lipid reserves to fuel biomass production and cell growth. We further demonstrated that this density-associated lipid droplet dynamic is largely conserved among Mtb isolates. Together, these findings support the hypothesis that under permissive conditions, Mtb utilizes lipid droplets as a carbon reservoir to regulate its growth.

Our in-depth analysis of culture density-feature associations also revealed an intriguing, lineage-defined phenotypic heterogeneity regarding DAPI staining. DAPI is a widely used DNA staining dye that is thought to freely diffuse across the plasma membrane (40). In gram-negative bacteria like *E. coli* and gram-positive bacteria such as *Staphylococcus aureus*, DAPI staining typically reaches equilibrium within 10 minutes and exhibits uniform distribution across cells (41). However, we found that in L2 and L4 isolates, a subpopulation of cells remained largely refractory to DAPI staining, even after prolonged incubation for several hours in the presence of high concentrations of DAPI. Moreover, the fraction of DAPI-null cells increases with culture density, suggesting a progressive shift in the physiological state of these otherwise genetically homogeneous cells. By contrast, L1 isolates exhibited consistent DAPI staining across different culture densities, lacking the staining heterogeneity observed in L2 and L4 isolates. One of the structural hallmarks of mycobacterial cells is their covalently crosslinked, multi-layered cell envelope, which serves as a formidable barrier against many hydrophilic compounds. Given our analyses and the chemical properties of DAPI, it is reasonable to speculate that the heterogeneous DAPI staining pattern in L2 and L4 isolates reflects intrinsic cellular variations in cell wall composition—a feature absent in L1 isolates. Such pronounced cell-to-cell differences in permeability may contribute to differential drug susceptibility profiles of clinical strains. However, the molecular mechanisms underlying this phenomenon and its clinical significance remain unclear and warrant further investigation.

Our study also revealed that, like *E. coli* and other rod-shaped bacteria (42, 43), Mtb undergoes reductive cell division as cell density increases. However, the rate of this monotonic decline in cell length varies significantly among isolates and is associated with specific Mtb subclades . A potential explanation for density-associated cell size dynamics is that, as bacteria deplete available carbon sources and density increases, the equilibrium between cell growth and cytokinesis shifts in a predictable, monotonic fashion. Based on this, it is possible that the homoplastic 50557T mutation in *ino1* alters how Mtb perceives the growth-division equilibrium, leading to a premature collapse in cell size at a much lower cell density.

The phylogenetic distribution of 50557T mutation across our global Mtb genome collections indicates that in L1, L2, and especially L4, this mutation is associated with recent expansion of specific clades (Fig. 6D), although the mechanistic association between the expansion of these 50557T variant clades and the phenotypic changes this mutation confers remains unclear. Notably, in another recent study, we applied BCP to Mtb isolates circulating in Lima, Peru, and established unexpected associations between an increased F420 autofluorescence and a highly transmissible subclade of L2 strains (g2g-L2), which locally emerged in Peru around 60 years ago but rapidly expanded *in situ* (26). This phenotype was further investigated and traced to a lineage-specific g2g-L2 mutation in *trxB2* (T2B), which was experimentally validated to enhance thioredoxin reductase activity (26). Taken together, our present and previous analyses revealed that the cytological landscape—on top of the culture density associated dynamics and within-strain heterogeneity—is indeed shaped by the phylogenetic structure of Mtb clinical strains and can be leveraged as a powerful proxy for studying Mtb clinical evolution.

### Limitations

Our study focused primarily on developing an accessible cytological profiling pipeline for *Mtb*, a BSL-3 pathogen. While this pipeline may link specific *Mtb* cellular features to genetic variants at the strain, clade, or lineage level, challenges remain in experimentally validating these associations, as such work requires labor-intensive genetic manipulation. While our system is optimized to generate comprehensive single-*Mtb* profiles, this study primarily focused on strain-level feature dynamics and only briefly explored within-strain variation across growth phases. Indeed, our primary analysis unveiled remarkable phenotypic heterogeneity of cells from the same strain. In addition to the aforementioned DAPI staining variations, another noteworthy heterogeneous feature is the autofluorescence of oxidized F420. As illustrated in Fig. 1B and 3B, and Fig. 4A, while most cells manifest diffused F420 signals, at lower culture densities, a fraction of cells show punctate F420 “granules” along the cell body. As F420 is a critical cofactor for a broad panel of metabolic enzymes, we speculate that F420 foci formation was due to the intracellular aggregation of these enzymes, which might reflect its functional compartmentalization within the cytoplasm of this subgroup of cells. Nevertheless, in the present study, we did not identify physiological or genetic correlates that could reliably explain the cause of F420 aggregation and its functional impact. We anticipate that a single-cell feature-driven image analysis—in conjunction with single-bacterium RNA sequencing—could yield critical insights into the physiological basis of Mtb phenotypic heterogeneity and its underlying evolutionary trajectories. Another limitation is that for many of the non-morphological features, their physiological correlates remain unclear, as does their relevance to Mtb transmission, disease manifestation, and the evolution of drug resistance in real-world settings (44). Drawing connections between these key phenotypes will require not only applying this imaging approach to patient-derived samples—such as sputum, bronchoalveolar lavage fluid (BALF), and other Mtb-containing specimens—but also integrating imaging data with complementary modalities such as transcriptomics. We look forward to addressing these technical limitations in future work.

In conclusion, this work presents a high-throughput microscopy platform that captures Mtb’s cytological dynamics across growth phases and lineages. We identified a convergent “small cell” phenotype linked to an *ino1* mutation, demonstrating the power of this approach in uncovering evolutionary traits. This method provides a scalable framework for studying drug responses, adaptation, and within-host heterogeneity, with future potential for expansion as new probes become available.

## MATERIALS AND METHODS

### Bacterial strains and culture conditions

A total of 64 Mtb clinical isolates were selected for this study, including 63 strains from a previously characterized collection in Ho Chi Minh City, Vietnam (33), and the laboratory reference strain H37Rv. These isolates represent a diverse set of Mtb lineages, including 15 lineage 1 (L1) strains, 40 lineage 2 (L2) strains, and 9 lineage 4 (L4) strains. Mtb cultures were grown in Middlebrook 7H9 broth supplemented with 10% oleic acid-albumin-dextrose-catalase (OADC) and 0.05% Tween-80 at 37°C with constant agitation (60 rpm). Mtb cultures were inoculated at an initial optical density at 600 nm (OD_600_) of 0.01 and grown to various OD_600_ values corresponding to different growth phases: early log (OD_600_ ~0.2), late log (OD_600_ ~0.8), early stationary (OD_600_ ~1.2), and late stationary (OD_600_ ~2.0). Peptidoglycan labeling was achieved by supplementing NADA-green (3-[(7-nitro-2,1,3-benzoxadiazol-4-yl)amino]-D alanine hydrochloride, biotechne, Cat. #6648) at a final concentration of 25 µM. *M. smegmatis* reporter strains were grown in Middlebrook 7H9 broth supplemented with 10% albumin-dextrose-catalase (ADC), 0.05% Tween-80, and 25 µg/mL kanamycin at 37°C with constant agitation (180 rpm).

### Molecular cloning and promoter reporter assay

To create the *P_ino1-AS_* promoter reporter strains, we first amplified the upstream fragment of the *P_ino1-AS_* promoter using JHIMCAS_87 (5′-CGGTCGGAACCCTCGCCGTCTAGAactcgcgcttcagattcgaag-3′) and JHIMCAS_93 (5′-GGGTAcccatcgtcggtgatg-3′), and the downstream fragment with corresponding single-nucleotide variations using JHIMCAS_89–92 (5′-tcaccgacgatgggTACCCNggcgtcggtgaacttcttgg-3′) and JHIMCAS_88 (5′-ATGtatatctccttcttTTAATTAAcggtgttcatcgcctccgac-3′), where “N” represents the four nucleotides T/C/G/A for primers JHIMCAS_89–92, respectively. Purified *M. tuberculosis* H37Rv genomic DNA was used as PCR template. The upstream and downstream fragments were fused by overlapping PCR using primers JHIMCAS_87 and JHIMCAS_88 and then column purified. The destination plasmid, pLJZ001, is a shuttle plasmid containing a UV15tetO promoter, a downstream codon-optimized mScarlet3 (45), and a L5 phage integration cassette (Fig. S8A). pJZ001 was cut using XbaI and PacI to excise the UV15tetO promoter, and the four *P_ino1-AS_* promoter variants were subcloned into the linearized plasmid via Gibson assembly. Sanger sequencing-verified reporter plasmids were transformed into wild-type *M. smegmatis* mc^2^155 via electroporation and selected on LB agar containing 25 µg/mL kanamycin. Three colonies were picked from each transformant as biological replicas and inoculated into 7H9 liquid broth containing kanamycin. Serial dilutions (1:200, 1:400, 1:800, 1:1,600, 1:3,200) of stationary phase cultures were inoculated into a clear bottom 96-well plate and shaken at 600 rpm for nine hours at 37°C before OD_600_ and fluorescence (Ex/Em: 561/600 nm) quantification using a TECAN Infinite 200Pro desktop plate reader. All sequences of the plasmids involved in this study are available from our Zenodo deposit (https://doi.org/10.5281/zenodo.15477993).

### High-throughput cytological profiling

To systematically characterize single-cell morphological and physiological features, we developed an optimized high-throughput cytological profiling pipeline for Mtb. This pipeline involved (i) sample preparation with on-gel staining, (ii) multi-channel fluorescence microscopy imaging, and (iii) automated image analysis using a custom-built software package, MOMIA2.

#### Agarose molding system for bacterial immobilization

To enable consistent imaging, we adapted a previously described agarose molding system (25) to create immobilization pads. Briefly, a custom 96-well molding tool was designed and fabricated using commercial-grade 3D printing or repurposed from recyclable accessories of pipette tip boxes (VWR). Fresh 1.8% molten agarose was prepared as follows: 0.8 g of low-electroendosmosis agarose (SeaKem LE Agarose, Lonza, Cat. # 50004) was added to 40 mL 1× PBS and mixed by gentle agitation. The mixture was then subjected to multiple short (<5 seconds) medium-power microwave pulses until it turned transparent and contained no undissolved, translucent agarose chunks. The molted agarose was briefly cooled (<5 minutes) at room temperature, then supplemented with DAPI (Sigma, Cat. #D9542) and Nile Red (Sigma, Cat. #72485) to final concentrations of 25 µg/mL and 100 ng/mL, respectively. The gel-containing falcon tube was rotated five to eight times and then slowly injected into the agarose mold using a serological pipettor. When the gel had flooded through the molding plane and filled all wells, a clean plate cover glass was placed on the gel and slid from one side to avoid large bubbles while sealing the top of the gel.

#### Bacterial fixation

Mtb cultures were harvested at different growth phases and mixed with equal volumes of 4% paraformaldehyde (PFA). The mixtures were covered with foil and incubated for one hour at room temperature. The fixed samples were decontaminated using high concentrations of Vesphene (Steris Cat. #647508) or bleach (Curr. Tech. Inc. Cat. #BRSPRAY16) before removal from the BSL-3 lab. While we recommend processing the samples for microscopy immediately after fixation, we found that prolonged fixation for up to a week had only a negligible impact on cytological profiles.

#### Chemical declumping

Chemical declumping was achieved through the following procedures: fixed cells were spun down at 3,000 × *g* for 5–10 minutes, and their supernatants were removed. Fresh declumping solution was prepared by adding 10 µL of xylene:heptane (2:1 [vol/vol]) mixture to 1 mL of quenching solution (100 mM Tris-HCl, pH = 7.5, 1% Triton X-100 [wt/vol]) and vortexing vigorously until the mixture was fully emulsified and turned cloudy. Each cell pellet was immediately resuspended using 100–200 µL of freshly prepared and emulsified declumping solution, incubated at room temperature for one minute, then spun down at 3,000 × *g* for five minutes. The pellets were then washed once with PBSTx solution (1× PBS supplemented with 0.1% Triton X-100) and resuspended with 1/5 to 1/100 its original volume, depending on the input cell density.

#### On-gel staining

On-gel staining was carried out by seeding 0.5–1 µL of de-clumped cell suspension onto the 96-well arrayed cover-glass surface (plate A attached to) and then flipping plate B to sandwich the cells between agarose and cover glass. The assembled imaging plate was incubated at 37°C for 30 minutes to allow DAPI and Nile red staining to reach equilibrium before microscopy imaging.

#### Automated image acquisition

Automated image acquisition was performed on a Nikon Ti-E inverted widefield microscope equipped with a Plan Apo 100×, 1.45 NA objective (with an internal phase ring), a Nikon Perfect Focus system, and a Piezo Z drive motor. Image capture was conducted using an Andor Zyla 4.2 sCMOS camera, with system control via NIS Elements (v4.5). A Spectra-X 6-color LED light engine provided excitation for Nile Red, NADA, F420 autofluorescence, and DAPI using filters with center wavelengths and FWHM passbands of 550/15, 470/24, 440/20, and 395/25  nm, respectively. Emission from Nile Red, NADA, and DAPI was collected using a multi-bandpass filter set (passbands centered at 595/40, 515/30, and 435/26  nm), while F420 autofluorescence was captured using a separate emission filter (475/20  nm). A custom NIS-Elements JOBS script automated well localization, autofocus, and multi-positional acquisition across the four fluorescence channels and bright-field phase contrast images.

#### Image processing and feature extraction

Image processing and feature extraction were performed using MOMIA2, an enhanced version of our previously established microscopy image analysis tool optimized for mycobacteria research. MOMIA2 automatically imports raw Nikon microscopy data in .nd2 format and segments phase contrast images using either a custom-built masked local thresholding algorithm or pre-generated segmentation masks from external tools (e.g., Omnipose [46]). Following segmentation, individual particles are identified, and their binary masks, subpixel outlines, and centerlines are computed to facilitate both primary profiling (e.g., cell morphology and fluorescence intensities) and advanced analyses of subcellular organization (e.g., lipid droplet distribution and nucleoid compactness). In addition, MOMIA2 applies a suite of local filters to phase contrast images—including difference-of-Gaussian (DoG), Laplacian-of-Gaussian (LoG), and ratios-of-Gaussian (RoG)—to generate single-particle profiles that help determine whether particles are out-of-focus or represent aggregates. Leveraging both primary and local features, a random forest classifier was trained on over 20,000 manually labeled particles to refine cell filtering. Finally, single-cell features from individual strain–OD_600_ pairs were aggregated to compute population-level statistics such as medians, interquartile ranges (IQR), and skewness. Sample-level feature statistics were listed in Table S1. The MOMIA2 package, Jupyter notebooks, and the source data included in this paper are available from our Zenodo deposit (https://doi.org/10.5281/zenodo.15477993).

### Modeling growth-dependent feature dynamics

To account for differences in growth rate and culture density across strains, raw feature values were normalized relative to optical density (OD_600_) using a LOWESS (locally weighted scatterplot smoothing) approach. First, a LOWESS model was applied to fit each numeric feature against log₂-transformed OD_600_ across all samples (strain-OD_600_ pairs). We evaluated multiple smoothing parameters (frac values of 0.25, 0.5, and 0.75) and ultimately selected a data set generated with a frac value of 0.3. In each case, the LOWESS trendline was computed and subtracted from the raw feature values to center them relative to the estimated trend. Subsequently, for each strain with at least three time points, a linear regression model was fitted to the LOWESS-normalized features as a function of log₂(OD_600_). This regression provided coefficients and intercepts for each feature and enabled the interpolation of feature values at defined OD_600_ levels (0.1, 0.4, 1.0, 2.0, and 4.0, Table S2). The interpolated values, along with the linear fit parameters for each feature, were compiled into a summary data set for subsequent analyses including feature selection, dimensionality reduction, and strain comparisons.

### Genome-wide association study (GWAS) and convergent evolution analysis

GWAS analyses were conducted using GEMMA software (v0.98.3) (47) to identify genomic variants associated with the “small cell” phenotype. The analysis was performed with the following parameters: a default missingness threshold of <0.05 and a minor allele frequency (MAF) cutoff of 0.01. Kinship matrices were incorporated to account for relatedness among samples. A linear mixed model was employed to control for the effects of MTB lineage, sublineage, and outbreak-associated population structure (48). To further validate the GWAS results, we conducted a phylogenetic convergence analysis to assess whether convergent evolutionary signals exist in the identified variants, thereby reducing potential bias from clonal population structure. For the convergent “small cell” phenotype, we identified a homoplastic mutation in ino1 (50557C>T) that occurred in multiple independent lineages. To explore its potential regulatory role, we analyzed RNA-Seq data from ~200 clinical Mtb strains and found that this mutation was uniquely associated with antisense RNA expression at the ino1 locus. dN/dS analysis for *ino1* was performed using the methods we described previously (49).

### RNA sequencing

Mtb was grown in a 1 mL volume in 24-well plates, covered with breathable film, at 37°C × 100 rpm in 7H9 media supplemented with 0.2% glycerol, 0.05% Tween-80, and 10% OADC. Strains were grown for eight days and back-diluted to achieve a final harvest OD_600_ of ~0.4. PFA was added to 2% to halt transcriptome dynamics. RNA-Seq libraries were prepared using a custom high-throughput library generation approach called Multiplex Taq-depleted Bacterial (MTB) RNA-Seq. Briefly, de-crosslinked RNA samples were barcoded using random heptamer reverse transcription with inline barcodes and 5′-adapters, then pooled for subsequent library steps. 3′-adapters were added via splint ligation, and rRNA depletion was conducted on single-stranded cDNA using Taq polymerase’s endonuclease activity for targeted cleavage of cDNA derived from rRNA. RNA-Seq data are uploaded to GEO (GSE294801), and full methods and strain information are available (48).

### Statistical analysis

All statistical analyses were conducted in Python (SciPy, statsmodels). Comparisons between lineages and sublineages were performed using Mann–Whitney *U* tests or Kruskal–Wallis ANOVA where appropriate. Correlations between OD_600_ and feature values were evaluated using Spearman’s rank correlation. UMAP dimensionality reduction was applied to visualize high-dimensional phenotypic data.

## Supplementary Material

Reviewer comments

## Data Availability

The full data set, including raw microscopy images, extracted feature data, and analysis scripts, will be made available upon request or is found in our Zenodo repository; the source code for MOMIA2 can be downloaded from our Zenodo deposit (10.5281/zenodo.15477993).

## SUPPLEMENTAL MATERIAL

Supplemental Material msystems.00972-25-s0001.pdf Figures S1-S8; captions for Tables S1 to S3.

Table S1 msystems.00972-25-s0002.xlsx Sample feature table.

Table S2 msystems.00972-25-s0003.xlsx LOWESS normalized, OD_600_ interpolated feature table.

Table S3 msystems.00972-25-s0004.xlsx Top 25 lineage-predictive features.

Movie S1 msystems.00972-25-s0005.mp4 Video tutorial for multi-well agarose cassette assembly.

OPEN PEER REVIEW reviewer-comments.pdf An accounting of the reviewer comments and feedback.

ASM does not own the copyrights to Supplemental Material that may be linked to, or accessed through, an article. The authors have granted ASM a non-exclusive, world-wide license to publish the Supplemental Material files. Please contact the corresponding author directly for reuse.
