# Supplementary material for: High-throughput cytological profiling uncovers genotype-phenotype associations in Mycobacterium tuberculosis clinical isolates
Source: mSystems. 2025 Oct 9;10(11):e00972-25. doi: 10.1128/msystems.00972-25 (PMC12625746; doi:10.1128/msystems.00972-25)
Supplement: Supplemental Material — Figures S1-S8; captions for Tables S1 to S3. [file msystems.00972-25-s0001.pdf]

Supplementary figure 1

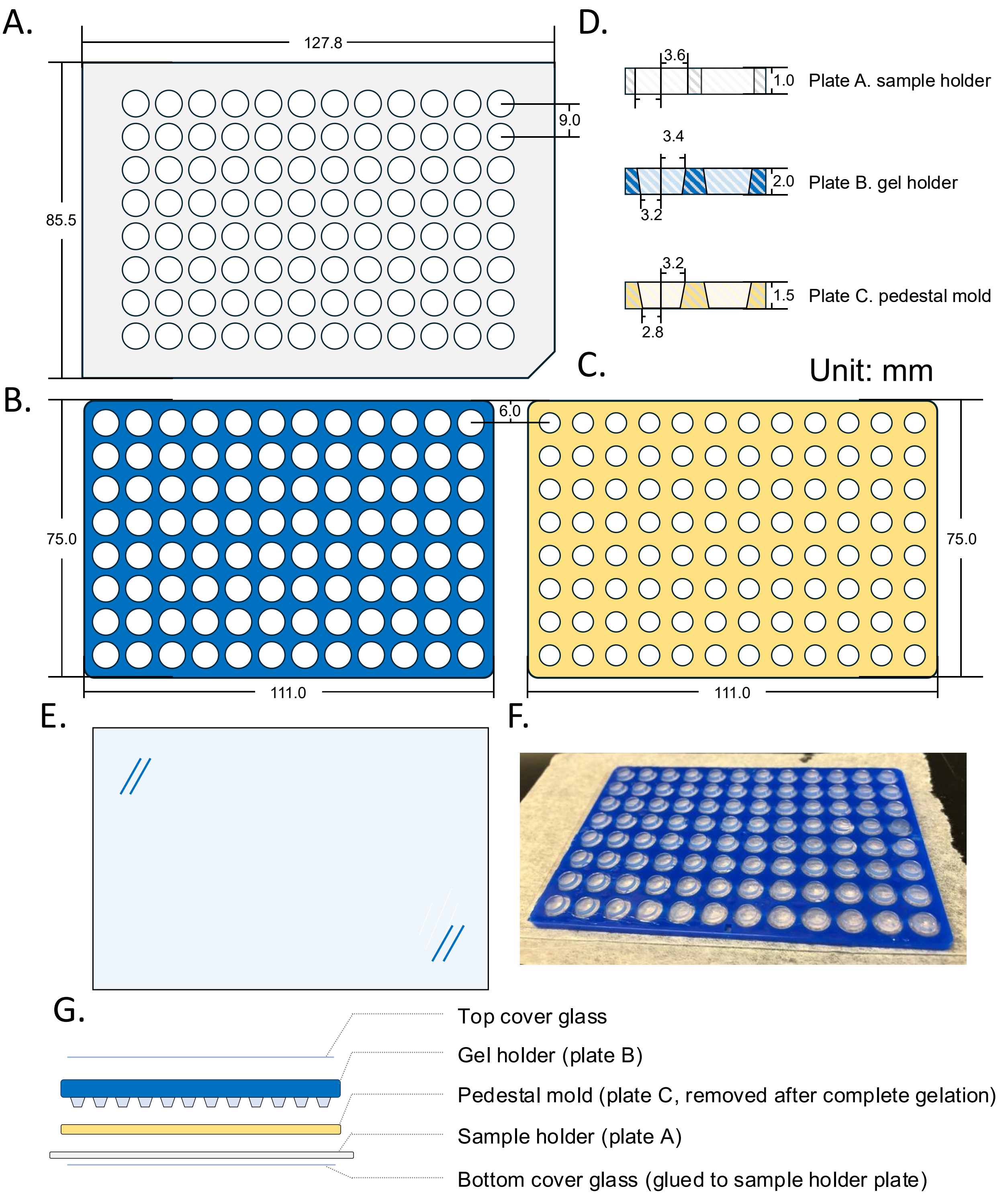

Supplementary Figure 1. Design and assembly of the multi-well agarose molding system for high-throughput bacterial cytological profiling. (**A-C**) Schematic representations of the three plates: Plate A (sample holder) serves as the base for sample positioning, Plate B (gel holder, blue) contains an array of wells for shaping the agarose gel, and Plate C (pedestal mold, yellow) creates raised agarose pedestals for bacterial immobilization. (**D**) Cross-sectional views show the dimensions of each plate in millimeters. (**E**) A glass cover is used to seal the gel holder during gelation. (**F**) A photograph of a fabricated gel holder (Plate B) with a fully formed agarose mold demonstrates the final structure. (**G**) The layered assembly consists of the gel holder (Plate B) placed on top of the sample holder (Plate A), with the pedestal mold (Plate C) removed after gelation. The system is enclosed by top and bottom cover glasses to ensure sample stability for imaging. This design enables uniform bacterial immobilization across multi-well plates, facilitating automated, high-throughput microscopy-based cytological profiling.

Supplementary figure 2

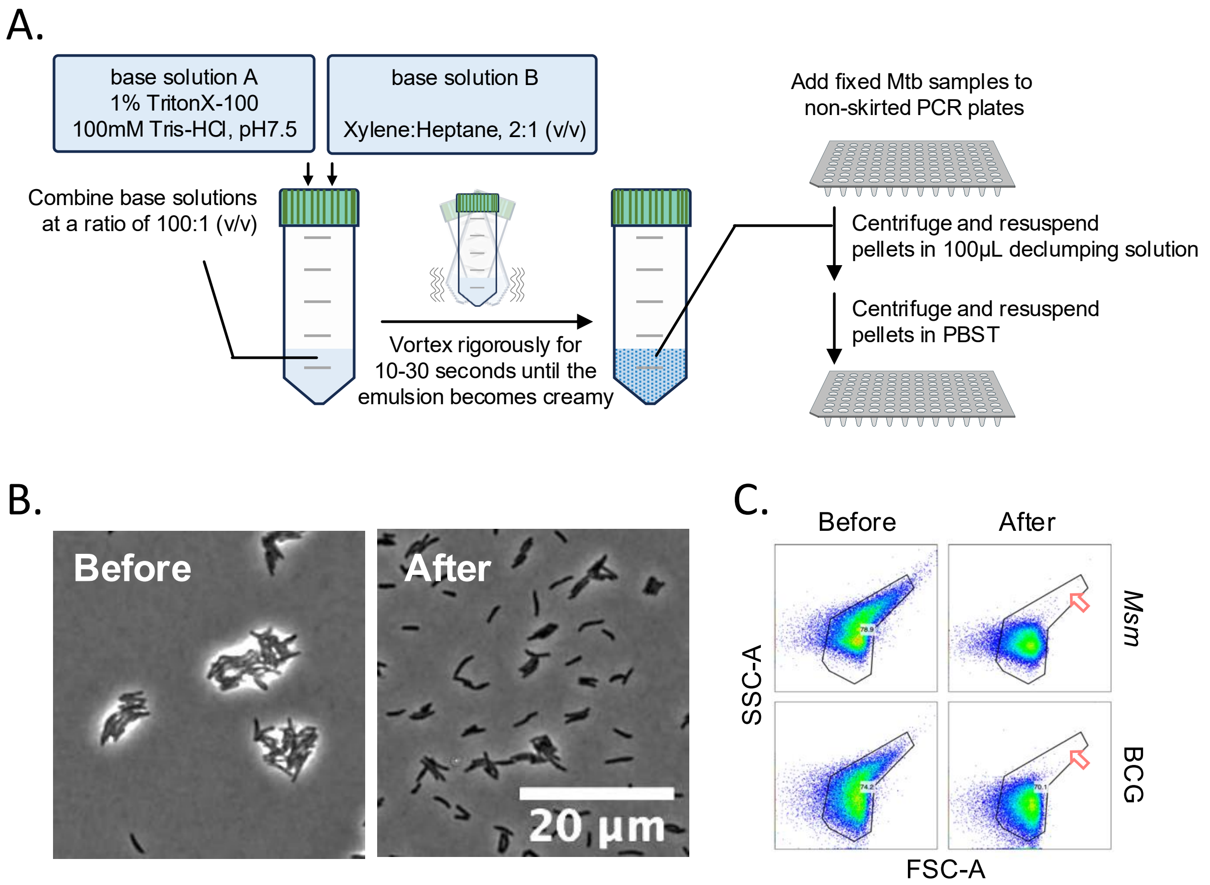

Supplementary Figure 2. Development and validation of the xylene-heptane emulsion-based bacterial declumping method. (**A**) Schematic workflow of the declumping procedure, where fixed Mtb samples are resuspended in a declumping solution composed of two base solutions: base solution A (1% Triton X-100, 100 mM Tris-HCl, pH 7.5) and base solution B (Xylene:Heptane, 2:1 v/v), mixed at a 100:1 ratio. The mixture is vortexed vigorously for 10–30 seconds until a creamy emulsion forms, followed by centrifugation and resuspension in PBST for downstream processing. (**B**) Phase-contrast microscopy images show bacterial aggregates before (left) and after (right) treatment, demonstrating effective dispersion into a more uniform single-cell suspension (scale bar: 20 µm). (**C**) Flow cytometry analysis of M. smegmatis (Msm) and Mycobacterium bovis BCG before (left) and after (right) declumping, where the shift in scatter patterns confirms successful reduction of cell aggregates.

Supplementary figure 3

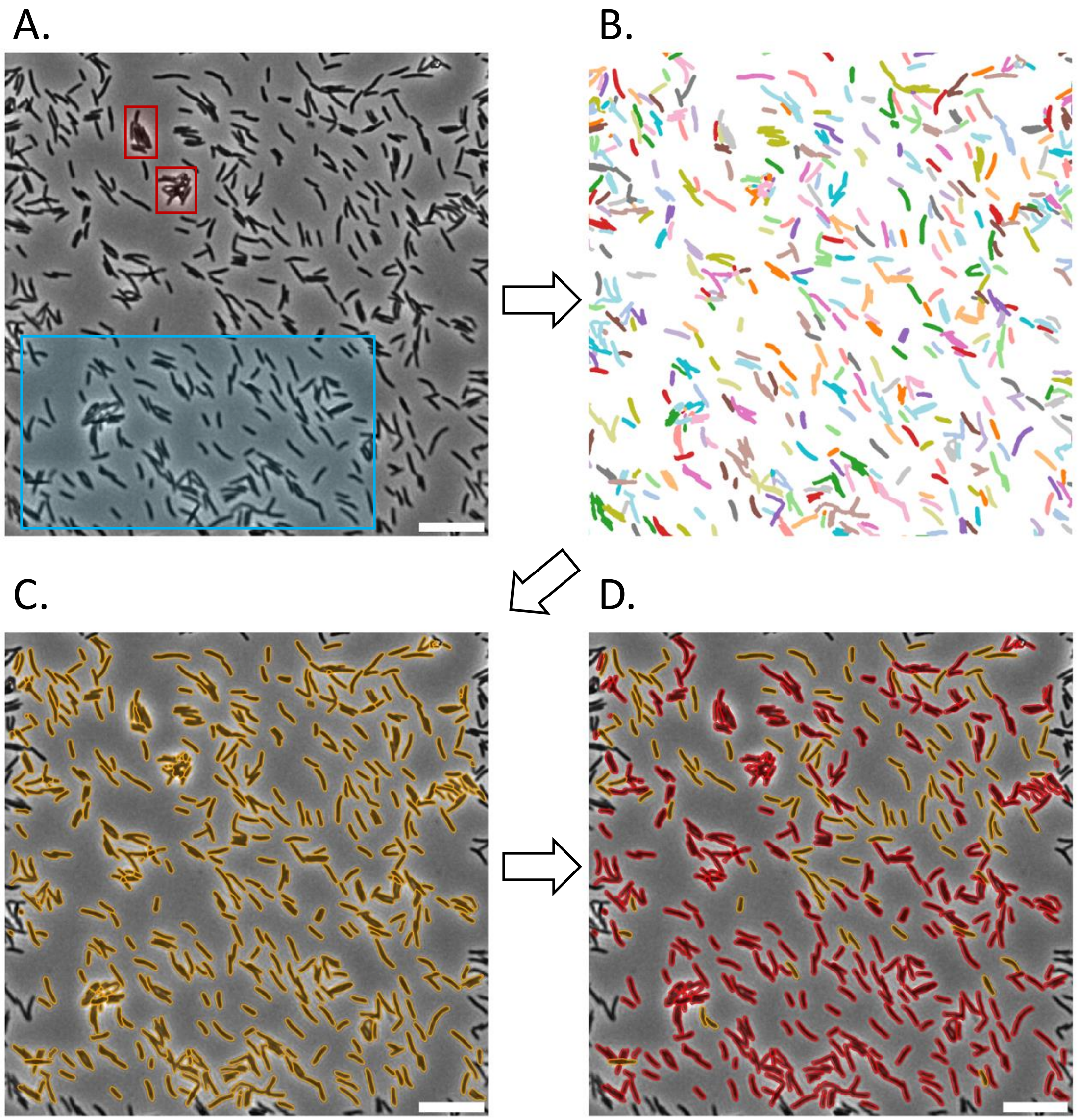

Supplementary Figure 3. Automated image processing and segmentation for single-cell analysis of Mtb. (**A**) Phase-contrast microscopy image of Mtb cells before processing, highlighting regions with bacterial aggregates (red boxes) and an area losing focus(blue box). (**B**) Segmentation of individual bacterial cells using a trained algorithm, with each cell assigned a unique color to visualize boundaries. (**C**) Identification of a subpopulation based on specific morphological criteria (highlighted in yellow). (**D**) Further classification of subpopulations, with an additional group of bacteria highlighted in red, allowing for downstream phenotype-based analyses. Scale bars: 5 μm.

Supplementary figure 4

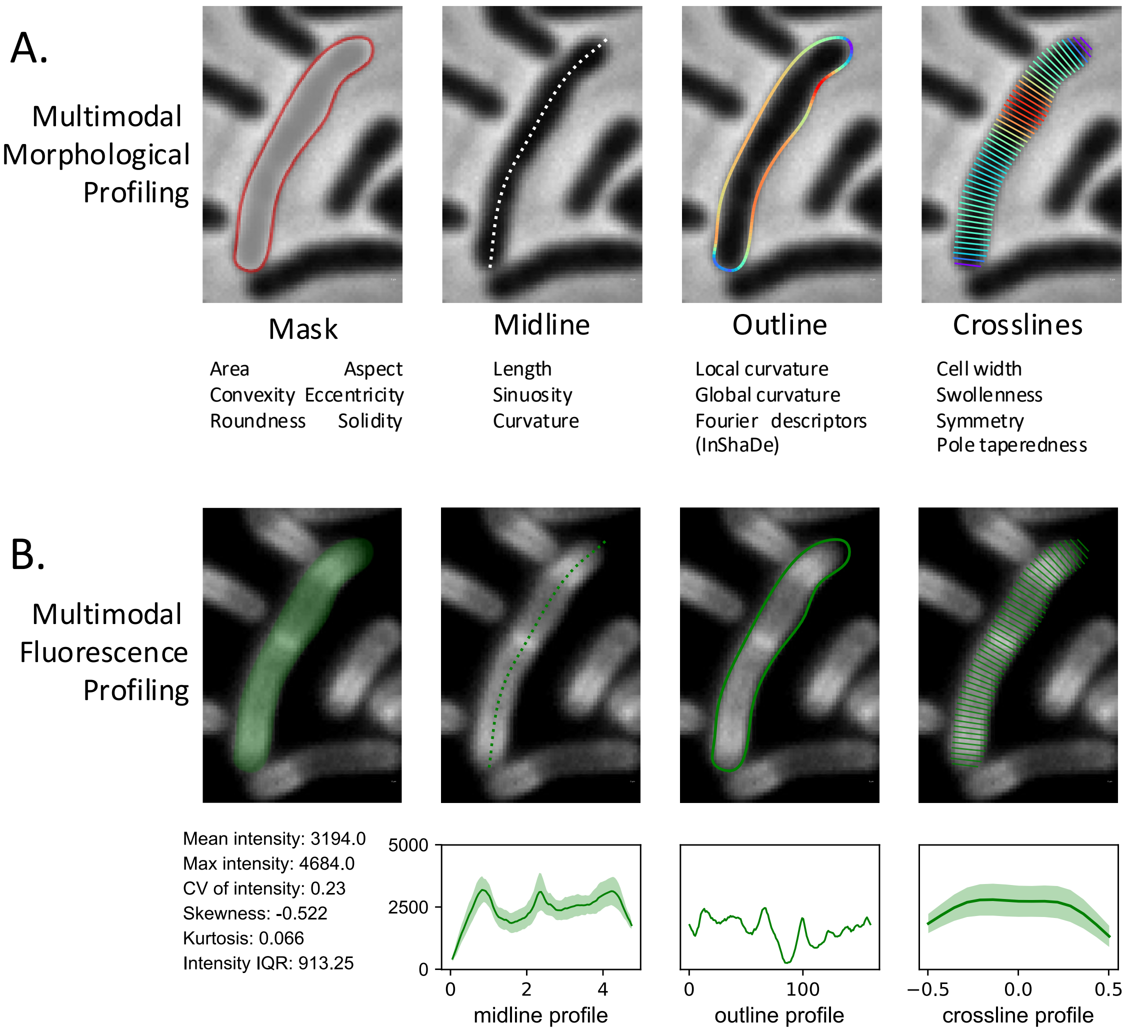

Supplementary Figure 4. Multimodal morphological and fluorescence profiling of Mtb at the single-cell level. (**A**) Multimodal morphological profiling includes: Mask (cell segmentation and shape features such as area, aspect ratio, convexity, roundness, and solidity), Midline (length, sinuosity, and curvature measurements), Outline (local and global curvature analysis using Fourier descriptors), and Crosslines (cell width, symmetry, pole tapering, and swelling distribution along the cell body). (**B**) Multimodal fluorescence profiling integrates intensity-based measurements along the midline, outline, and crossline profiles, enabling spatially resolved fluorescence quantification. Example fluorescence metrics include mean and maximum intensity, coefficient of variation (CV), skewness, kurtosis, and interquartile range (IQR). Fluorescence intensity distribution plots further demonstrate how fluorescence signals are spatially organized within individual cells, supporting detailed phenotypic characterization.

Supplementary figure 5

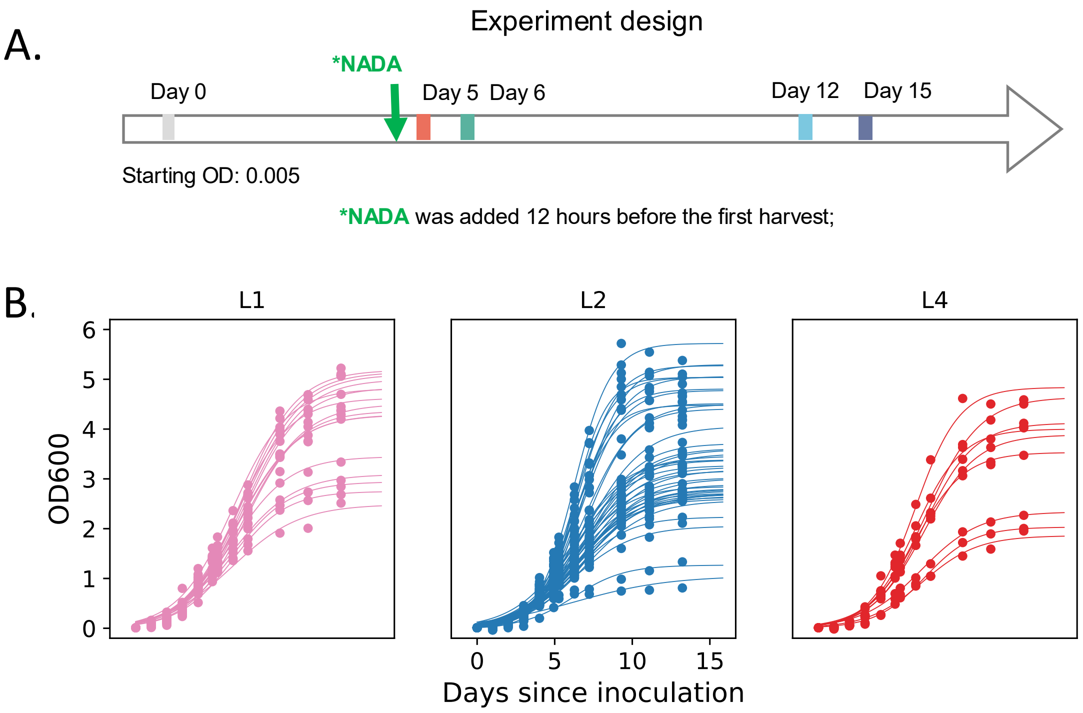

Supplementary Figure 5. Experimental design and growth dynamics of Mtb lineages. (**A**) Schematic representation of the experimental design. *Mtb* cultures were initiated at an OD600 of 0.005 and sampled at multiple time points (Days 6, 12, and 15). NADA, a fluorescent D-amino acid used for peptidoglycan labeling, was added 12 hours before the first harvest to allow incorporation into actively growing cells. (**B**) Growth curves of *Mtb* clinical isolates from lineage 1 (L1, pink), lineage 2 (L2, blue), and lineage 4 (L4, red), showing OD600 measurements over time. Each dot represents an individual measurement, and fitted curves illustrate strain-specific growth trajectories. The data highlight lineage-specific differences in growth kinetics and heterogeneity in bacterial replication rates within each lineage.

Supplementary figure 6

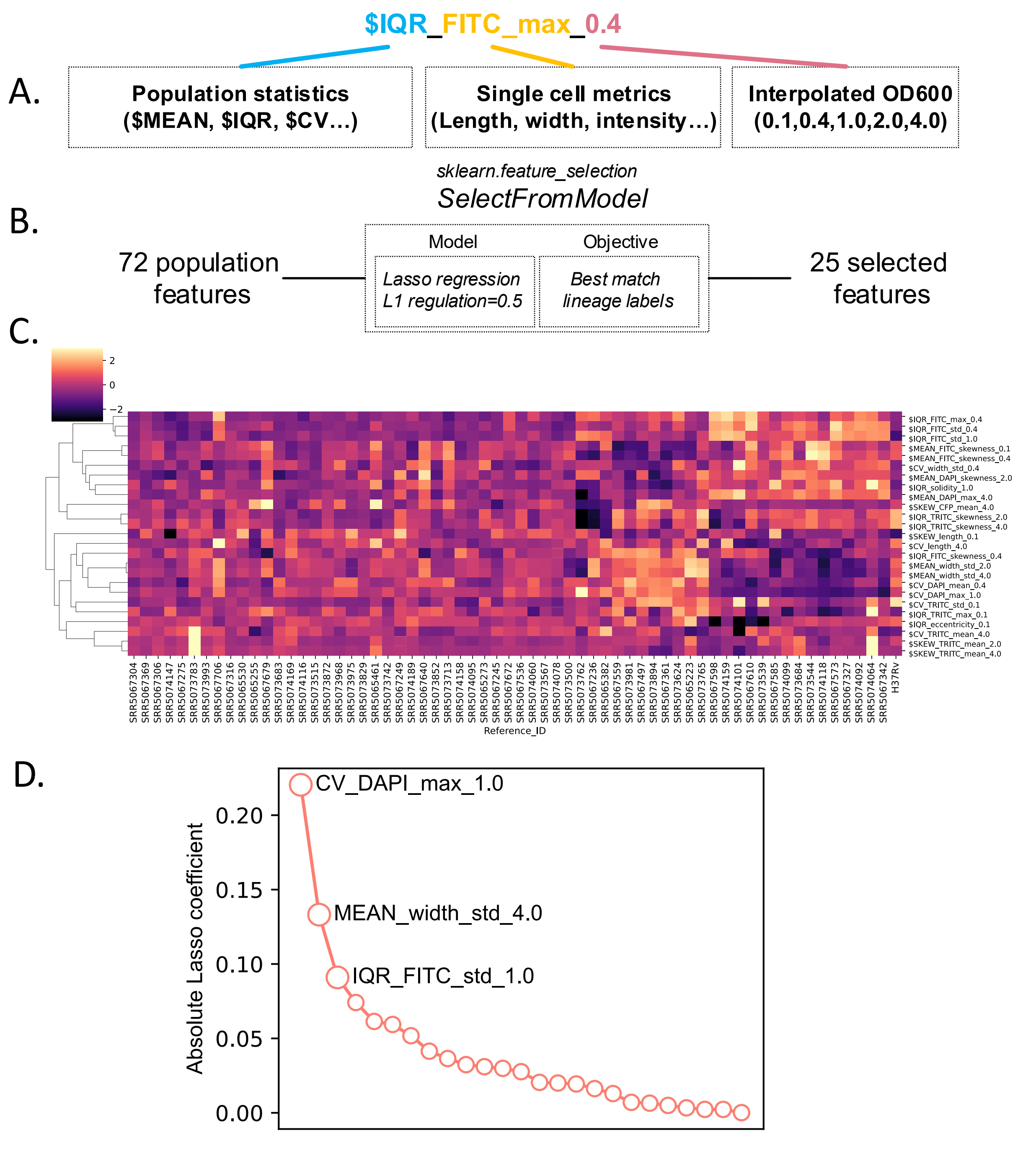

Supplementary Figure 6. Feature selection for lineage-associated bacterial traits using Lasso regression. (**A**) Overview of feature categories used in the analysis, including population-level statistics (e.g., mean, interquartile range (IQR), and coefficient of variation (CV)), single-cell morphological and intensity-based metrics (e.g., length, width, fluorescence intensity), and interpolated OD600 values corresponding to different growth phases. (**B**) Feature selection pipeline using Lasso regression with L1 regularization (α = 0.5). A total of 72 population-level features were assessed, and the model was optimized to best match lineage labels. The *SelectFromModel* function from *sklearn.feature_selection* was applied to identify the most informative features, yielding a final set of 25 selected features. (**C**) Heatmap representation of the selected 25 features across different *Mtb* strains, showing hierarchical clustering of phenotypic profiles, with color intensity indicating scaled feature values. This analysis highlights key lineage-associated traits and their variability across the dataset. (D) A scatter-line plot depicting the top 25 lineage-predicting features ranked by Lasso coefficients with the three most influential features highlighted.

Supplementary figure 7

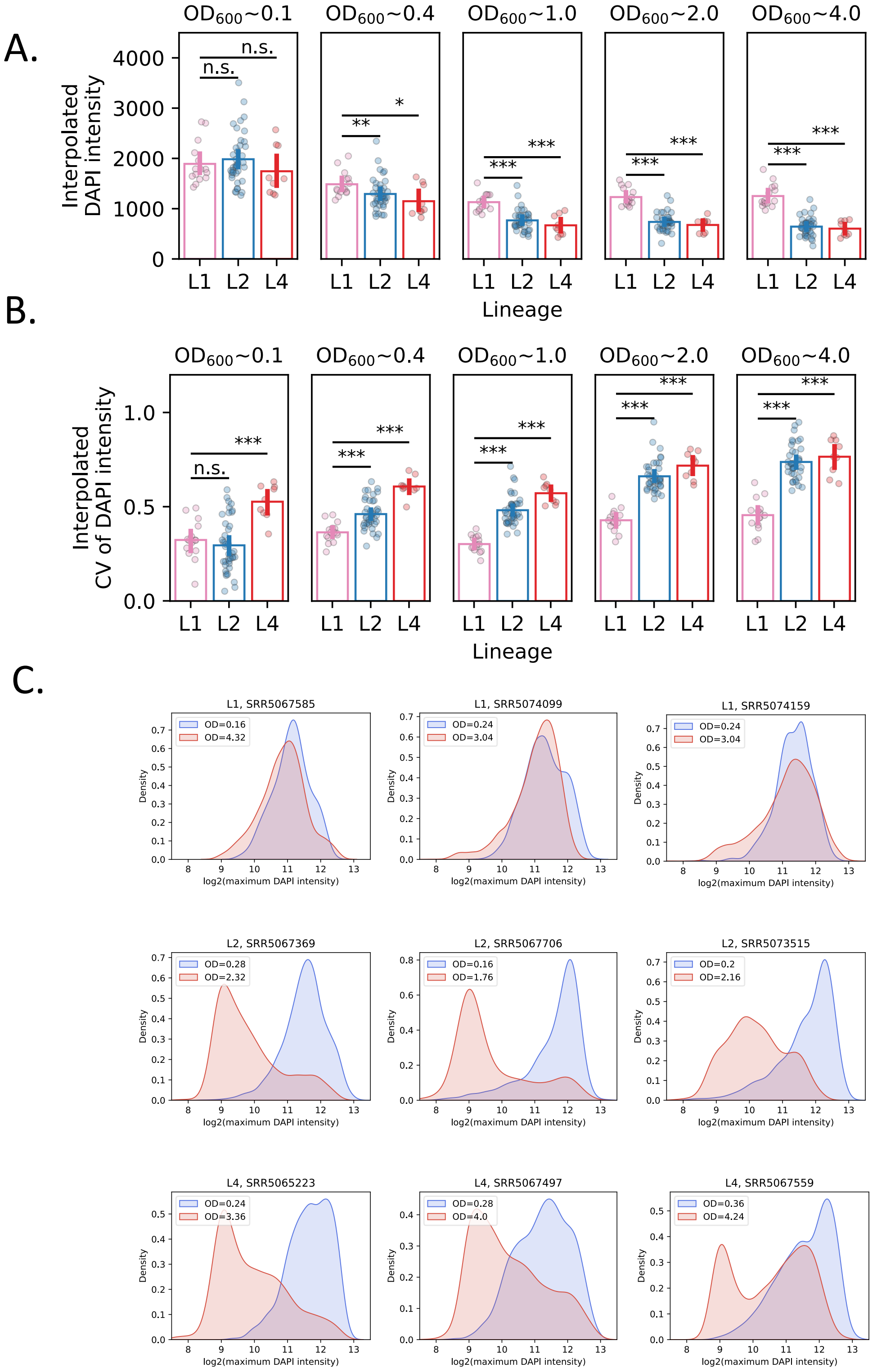

Supplementary Figure 7. Growth phase-dependent variability in DNA stainability across Mtb lineages.
(A-B) Bar charts displaying the LOWESS-normalized and OD interpolated average DAPI intensities **(A)** and coefficient of variations of DAPI intensity **(B)** of isolates from L1, L2, and L4. Error bars indicate the 95% confidence intervals. Significance between groups is estimated using Mann-Whitney-U test and indicated by asterisks: *** for $p < 0.001$, ** for $p < 0.01$, * for $p < 0.05$, and n.s. for non-significant differences.
(C) Density plots showing the distribution of log_2-transformed maximum DAPI fluorescence intensity in representative *Mtb* strains from lineages 1 (L1), 2 (L2), and 4 (L4) at low OD (blue) and high OD (red) states.

Supplementary figure 8

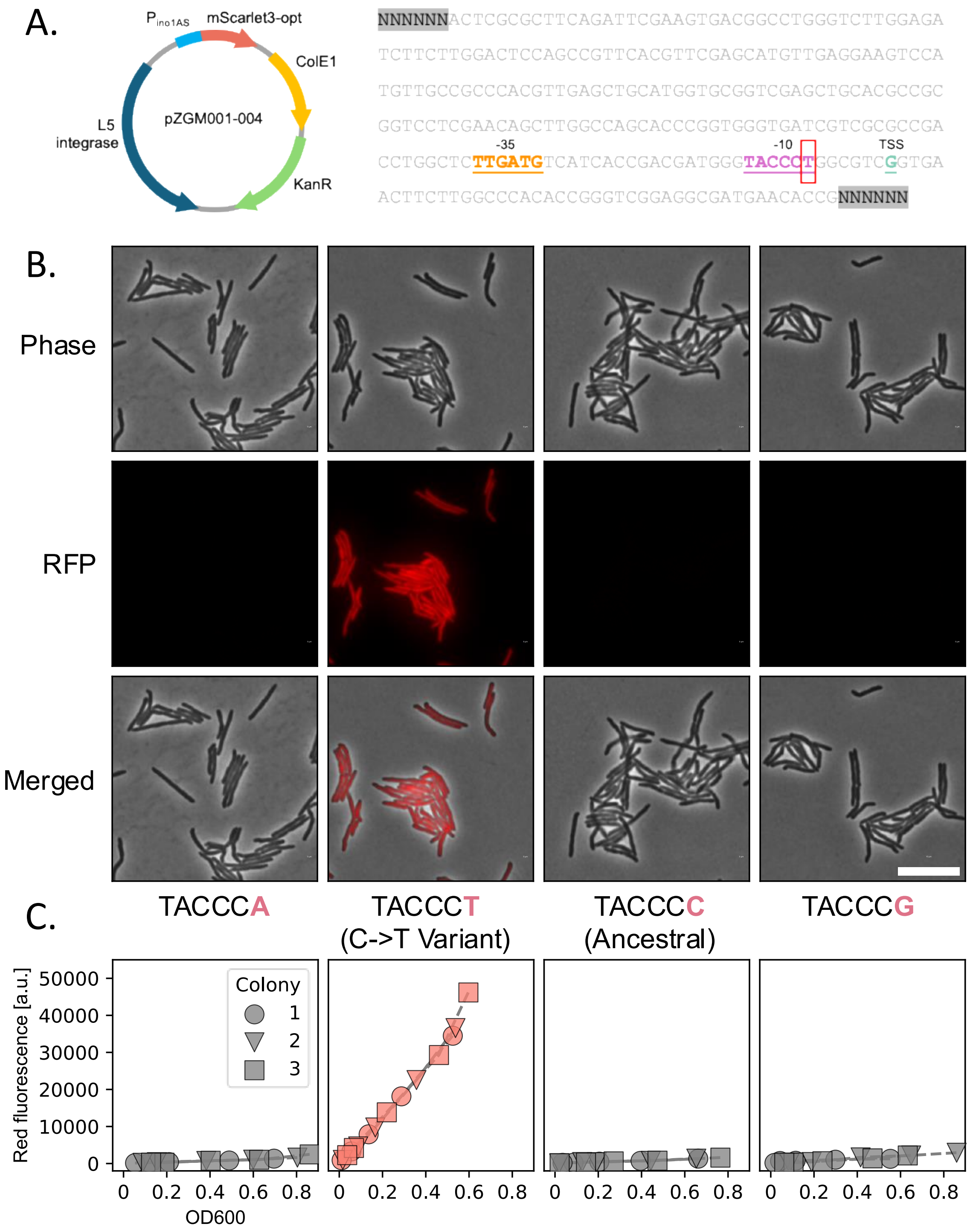

Supplementary Figure 8. Validation of allele-specific gene expression from the cryptic *ino1* antisense promoter using a fluorescent protein reporter system.

(A) Schematic of *ino1* antisense promoter reporters. Left: a plasmid map denoting the fusion construct of *ino1* antisense promoter (P_{ino1AS}) variants and a codon-optimized mScarlet3 gene. Right: a sequence map of the manually selected *ino1* antisense promoter (P_{ino1AS}) fragment. Gray-shaded random nucleotides (N) denotes the adjacent plasmid backbone and the red box highlights the 50557 locus, which constitutes the last nucleotide of the putative -10 consensus of P_{ino1AS} and were mutated to all four nucleotides to yield the promoter variants.

(B-C) 50557T but not other single-nucleotide variants drives strong expression of its downstream genes. **(B)** Representative microscopy images of *M. smegmatis* expressing mScarlet3 from the four single-nucleotide P_{ino1AS} variants. Scaler bar: 5μm. **(C)** Three colonies from each M. smegmatis transformant harboring a P_{ino1AS} variant reporter were serial diluted, cultured for 9 hours, then measured for OD_{600} and red fluorescence. Single colonies were represented by different marker shapes and their OD_{600} - fluorescence dynamics were separately plotted as grey dashed lines.

Table S1. Sample feature table

This table contains the filtered feature data for each strain at different optical densities (ODs). It includes annotation columns (e.g., Lineage, Reference_ID) and the 72 feature columns (marked by $ sign) used for strain comparison. Each feature represent the specific statistical characterization (e.g., $MEAN, $CV) of cellular metrics (e.g., length, DAPI_mean) across a given sample.

Table S2. LOWESS normalized, OD_{600} interpolated feature table.

This table presents the interpolated feature data, which is generated by interpolating the feature values at specific ODs (0.1, 0.4, 1.0, 2.0, and 4.0) using a linear model fitted on the LOWESS-normalized data.

Table S3. Top 25 lineage-predictive features.

This table lists the names and descriptions of the 25 features most predictive of Mtb lineage. We identified these features by applying a Lasso regression model to the LOWESS-normalized, OD-interpolated data and ranking them by feature importance.
