# Supplementary material for: High-throughput cytological profiling uncovers genotype-phenotype associations in Mycobacterium tuberculosis clinical isolates
Source: mSystems. 2025 Oct 9;10(11):e00972-25. doi: 10.1128/msystems.00972-25 (PMC12625746; doi:10.1128/msystems.00972-25)
Supplement: Reviewer comments [file reviewer-comments.pdf]

AMERICAN SOCIETY FOR MICROBIOLOGY | mSystems®

High-throughput Cytological Profiling Uncovers Genotype-Phenotype Associations in *Mycobacterium tuberculosis* Clinical Isolates

Qingyun Liu, Yue Liu, Ruiyuan Liu, Peter Culviner, Xin Wang, Ian Wolf, Ken Chen, Yiwang Chen, Yi Xiao, Guiming Zhang, Rongfeng Sun, Shoko Wakabayashi, Nicole Howard, Mingyu Gan, Eric Rubin, Sarah Fortune, and Junhao Zhu

Corresponding Author(s): Junhao Zhu, Institute of Microbiology Chinese Academy of Sciences

Review Timeline:

Submission Date:	June 30, 2025
Editorial Decision:	August 5, 2025
Revision Received:	September 16, 2025
Accepted:	September 19, 2025

Editor: Mark Mandel

Reviewer(s): Disclosure of reviewer identity is with reference to reviewer comments included in decision letter(s). The following individuals involved in review of your submission have agreed to reveal their identity: Jianping Xie (Reviewer #2)

Transaction Report:

(Note: With the exception of the correction of typographical or spelling errors that could be a source of ambiguity, letters and reports are not edited. The original formatting of letters and referee reports may not be reflected in this compilation.)

DOI: https://doi.org/10.1128/msystems.00972-25

Re: mSystems00972-25 (**High-throughput Cytological Profiling Uncovers Genotype-Phenotype Associations in *Mycobacterium tuberculosis* Clinical Isolates**)

Dear Dr. Junhao Zhu:

Thank you for the privilege of reviewing your work. Below you will find instructions from the mSystems editorial office and the reviewer comments. You will see that both reviewers found the manuscript to be exceptionally strong with only minor comments on the manuscript. The reviewer comments can be satisfied with adjustments in the figures and text without the need for any new experiments.

Please return the manuscript within 60 days; if you cannot complete the modification within this time period, please contact me. If you do not wish to modify the manuscript and prefer to submit it to another journal, notify me immediately so that the manuscript may be formally withdrawn from consideration by mSystems.

Revision Guidelines
To submit your modified manuscript, log into the submission site at https://msystems.msubmit.net/cgi-bin/main.plex. Go to Author Tasks and click the appropriate manuscript title to begin. The information you entered when you first submitted the paper will be displayed; update this as necessary. Note the following requirements:

• Upload point-by-point responses to the issues raised by the reviewers in a file named "Response to Reviewers," NOT in your cover letter.
• Upload a compare copy of the manuscript (without figures) as a "Marked-Up Manuscript" file.
• Upload a clean .DOC/.DOCX version of the revised manuscript and remove the previous version.
• Each figure must be uploaded as a separate, editable, high-resolution file (TIFF or EPS preferred), and any multipanel figures must be assembled into one file.
• Any supplemental material intended for posting by ASM should be uploaded with their legends separate from the main manuscript. You can combine all supplemental material into one file (preferred) or split it into a maximum of 10 files with all associated legends included.

For complete guidelines on revision requirements, see our Submission and Review Process webpage. Submission of a paper that does not conform to guidelines may delay acceptance of your manuscript.

Data availability: ASM policy requires that data be available to the public upon online posting of the article, so please verify all links to sequence records, if present, and make sure that each number retrieves the full record of the data. If a new accession number is not linked or a link is broken, provide mSystems production staff with the correct URL for the record. If the accession numbers for new data are not publicly accessible before the expected online posting of the article, publication may be delayed; please contact production staff (mSystems@asmusa.org) immediately with the expected release date.

Publication Fees: For information on publication fees and which article types are subject to charges, visit our website. If your manuscript is accepted for publication and any fees apply, you will be contacted separately about payment during the production process; please follow the instructions in that e-mail. Arrangements for payment must be made before your article is published.

ASM Membership: Corresponding authors may join or renew ASM membership to obtain discounts on publication fees. Need to upgrade your membership level? Please contact Customer Service at Service@asmusa.org.

The ASM Journals program strives for constant improvement in our submission and publication process. Please tell us how we can improve your experience by taking this quick Author Survey.

Thank you for submitting your paper to mSystems.

Sincerely,
Mark Mandel
Editor
mSystems

Reviewer #1 (Comments for the Author):

to map the cell morphological and physiological landscape of Mtb clinical isolates. They describe the technical details of how they imaged so many strains, and went into detail about the sophisticated image analysis. Much of the variation they find stems from different growth phases independent of the different lineages. Nevertheless, they identify a phenotypic difference in a few of their isolates, which might arise from a mutation causing an antisense transcript. While the authors don't confirm that this

mutation is causing the 'mini-cell' phenotype or explore the underlying mechanisms, I think that this manuscript represents an important advance that will be valuable to the field. I particularly appreciated the associated video, showing how they fabricated their 96-well devices. I suggest just a few small changes.

In bacteriology, a 'mini-cell' is one that is typically formed from a misplaced septa/chromosome and is missing DNA. The authors' use of the term here specifies something that they don't demonstrate, nor do I think it is consistent with their data. I recommend revising to something else - like 'small cell'

Fig 1B: size of scale bar missing.

Fig 1C: no y-axis labels . Do they all start at y=0?, no description of what the error is? Why is there no error associated with cell width?

Figure 2 - OD600 panel. I'm confused about the colors of the dots on the OD plot? What do the different colors indicate? The colors are similar to the ones indicating the different lineages and should be revised (or made uniform).

Figure 3B - size of scale bar missing. 3C - no units on the y-axis. Even if the units are arb. U. the y-axis label could be more descriptive to indicate mean intensity, or total intensity, etc. Why is the F420 fluorescence so punctate at the lower ODs?

Figure 4H - what is the green fluorescence?

Minor typos:

Line 60 - 'is' to 'are'. Mycobacteria not capitalized.

Line 110 - disperse to disperses and quench to quenches

Line 153 - 'incorporates'

Lines 156-157 - I recommend changing to the ...' clinical strains have different growth rates'

Lines 210 - coefficient of variation doesn't need to be capitalized

Reviewer #2 (Comments for the Author):

This manuscript presents an innovative high-throughput cytological profiling pipeline for Mycobacterium tuberculosis that significantly advances our ability to connect genetic variation with phenotypic consequences.While the study is methodologically robust and the conclusions are generally well-supported, some aspects require clarification and additional experimental validation to fully realize the potential impact of these discoveries.

1.The 50557 C>T mutation is not only potentially linked to antisense RNA expression, as emphasized by the authors, but also resides within the ino1 gene. Given that the mutation is under purifying selection in ino1, could it impair the protein's function and thereby contribute to the observed phenotype?

2.Since the authors did not validate the identified SNPs through genetic experiments to reproduce the phenotype changes, this limitation should be discussed.

3.How did the authors account for potential linkage disequilibrium between the identified GWAS hits and neighboring variants in their analysis?

4.The legend position in Fig. 6B should be adjusted, and statistical analysis should be provided for the violin plots.

5.Reporter gene assays are necessary to demonstrate changes in antisense RNA transcript levels resulting from the 50557 C>T mutation.

6.In the heatmap of Fig. 5A, the authors compare 25 different cytological features. However, it is unclear what these 25 features represent, as only five cytological features were mentioned in earlier results.

Responses to reviewers' comments

September 12, 2025

Manuscript ID: *mSystems00972-25*

Manuscript title: *High-throughput Cytological Profiling Uncovers Genotype-Phenotype Associations in Mycobacterium tuberculosis Clinical Isolates*

Reviewer #1 (Comments for the Author):

to map the cell morphological and physiological landscape of Mtb clinical isolates. They describe the technical details of how they imaged so many strains, and went into detail about the sophisticated image analysis. Much of the variation they find stems from different growth phases independent of the different lineages. Nevertheless, they identify a phenotypic difference in a few of their isolates, which might arise from a mutation causing an antisense transcript. While the authors don't confirm that this mutation is causing the 'mini-cell' phenotype or explore the underlying mechanisms, I think that this manuscript represents an important advance that will be valuable to the field. I particularly appreciated the associated video, showing how they fabricated their 96-well devices. I suggest just a few small changes.

Reply: Thank you for your very kind and insightful comments. We agree that our findings on the "small cell" phenotype and others are only early steps, and we hope that by sharing these observations and the techniques used to generate them, we can foster future studies into novel Mtb physiology by the wider community.

In bacteriology, a 'mini-cell' is one that is typically formed from a misplaced septa/chromosome and is missing DNA. The authors' use of the term here specifies something that they don't demonstrate, nor do I think it is consistent with their data. I recommend revising to something else - like 'small cell'

Reply: Thank you for pointing this out. We have changed it to "small cell" throughout the manuscript.

Fig 1B: size of scale bar missing.

Reply: We appreciate this catch and have now added scale bar sizes to Fig.1B legend.

Fig 1C: no y-axis labels . Do they all start at y=0?, no description of what the error is? Why is there no error associated with cell width?

Reply: Yes, all Y-axes in Fig.1C start at 0. To generate feature profiles along the cell axis, our analysis pipeline first finds the centerline then computes a series of equally spaced orthogonal lines along the centerline. For fluorescence data, the mean and standard deviation of data interpolated along each orthogonal line is measured and used to plot the fluorescence profile lines and shaded errors (one std from the mean) depicted in Fig.1C. For cell width, we simply take the distance between the two intersection points of each orthogonal line and the cell outline,

which is why there is no error associated with cell width. We apologize for the lack of clear explanation and have revised the figure panel and the legends accordingly.

Figure 2 - OD600 panel. I'm confused about the colors of the dots on the OD plot? What do the different colors indicate? The colors are similar to the ones indicating the different lineages and should be revised (or made uniform).

Reply: Thanks for the helpful comments. To avoid confusion, we have followed the reviewer's suggestion and adjusted the color to grey to differentiate it from lineage colors.

Figure 3B - size of scale bar missing. 3C - no units on the y-axis. Even if the units are arb. U. the y-axis label could be more descriptive to indicate mean intensity, or total intensity, etc. Why is the F420 fluorescence so punctate at the lower ODs?

Reply: We appologize this oversight and thanks for pointing this out. We have now included the scale bar in the revised figure and corected the legends accordingly.

We agree that the punctate F420 fluorescence is an intriguing observation. As you correctly noted, there is an inverse correlation between the formation of F420 puncta and culture density. We quantified this relationship using the cellular kurtosis of F420 fluorescence, where a higher kurtosis indicates a greater concentration of signal into puncta. This is depicted in Figure 4A and briefly explained on lines 184-187 of the revised manuscript. Notably, this finding is consistent with our previous work, where we observed F420 puncta in exponentially growing Mtb clinical isolates (PMID: 39613754). F420 is a deazaflavin coenzyme that Mtb produces in abundance and is utilized by a broad panel of metabolic enzymes (PMID: 20675471). Therefore, we hypothesize that during active growth, certain F420-dependent enzymes may undergo liquid-liquid phase separation to form membraneless metabolic compartments. This would enhance the efficiency of F420 utilization and regeneration, resulting in the observed aggregation. While we are actively exploring this phenomenon, a full investigation into its physiological basis is beyond the scope of the present study. We look forward to elaborating on this in future publications and have addressed this limitation on lines 368-377.

Figure 4H - what is the green fluorescence?

Reply: Thank you. We believe that you meant to query about Fig. 5H. The green fluorescence is NADA and we have now added description in the figure legend.

Minor typos:

Line 60 - 'is' to 'are'. Mycobacteria not capitalized.

Reply: Thank you! We have corrected them accordingly.

Line 110 - disperse to disperses and quench to quenches

Reply: Now changed to "disperses" and "quenches".

Line 153 - 'incorporates'

Reply: Now changed to "incorporates".

Lines 156-157 - I recommend changing to the ...' clinical strains have different growth rates'

Reply: Revised per your kind suggestion.

Lines 210 - coefficient of variation doesn't need to be capitalized

Reply: Now changed to lower cases.

Reviewer #2 (Comments for the Author):

This manuscript presents an innovative high-throughput cytological profiling pipeline for Mycobacterium tuberculosis that significantly advances our ability to connect genetic variation with phenotypic consequences.While the study is methodologically robust and the conclusions are generally well-supported, some aspects require clarification and additional experimental validation to fully realize the potential impact of these discoveries.

1.The 50557 C>T mutation is not only potentially linked to antisense RNA expression, as emphasized by the authors, but also resides within the ino1 gene. Given that the mutation is under purifying selection in ino1, could it impair the protein's function and thereby contribute to the observed phenotype?

Reply: Thank you for the insightful question. Indeed, apart from creating a cryptic promoter on the antisense strand of the coding gene ino1, 50557C>T mutation also changes the 190_{th} amino acid of Ino1 from Glycine to Arginine. Notably, there are two possible routes of SNP mutatgenesis which converts the ancestral Gly190 codon *GGG* to a proximal Arginine codon: *AGG* (50557C>T) or *CGG* (50557C>G), whereas to change to the four other Arginine codons (AGA/CGT/CGC/CGA), it would require the simultaneous mutation of two bases. Across our global Mtb strain collection (Fig. 6D), we did not find any 50557C>G variants nor other two-nucleotide variants, even though CGG is a much more frequently used Arginine codon compared to AGG (34% vs. 4% genome-wide usage frequency according to the Kazusa codon usage database https://www.kazusa.or.jp/codon/cgi-bin/showcodon.cgi?species=83332). Therefore, while we could not fully rull out the possibility that Ino1 protein sequence variation (G190R) contributes to the “small-cell” phenotype, the observed nucleotide bias implies that the selection underlying 50557C>T mutation is primarily associated with the non-coding function of the cryptic antisense promoter rather than the *ino1* coding sequence. We now include a short paragraph (lines 267-273) to address the above codon-bias analysis.

2.Since the authors did not validate the identified SNPs through genetic experiments to reproduce the phenotype changes, this limitation should be discussed.

Reply: Thank you. We agree that this is an important query that we did not address in the present manuscript. We have revised the discussion (lines 356-359) to briefly address this limitation.

3.How did the authors account for potential linkage disequilibrium between the identified GWAS hits and neighboring variants in their analysis?

Reply: Thank you for your comment. In our analysis, GEMMA's linear mixed model framework was used. By incorporating a genome-wide relatedness matrix, this approach accounts for background linkage disequilibrium (LD) and population structure, which is the commonly adopted strategy in previous GWAS of M. tuberculosis [PMID: 31086182, 29358649]. However, as the reviewer correctly points out, local LD cannot be fully removed by this framework. This explains why some sublineage-specific mutations still appeared among our hits. To minimize false positives, we (i) excluded all sublineage-specific mutations based on the phylogenetic structure, and (ii) relied on signals supported by convergent evolution to enhance robustness. We also note that more refined GWAS methods tailored for the highly clonal and extremely linked genome of *M. tuberculosis* are under development, but these are beyond the scope of the present study.

4.The legend position in Fig. 6B should be adjusted, and statistical analysis should be provided for the violin plots.

Reply: Thank you for pointing it out. We have changed the legend position to its correct form and have provided the statistical analysis results in the Fig.6B and its corresponding legend.

5.Reporter gene assays are necessary to demonstrate changes in antisense RNA transcript levels resulting from the 50557 C>T mutation.

Reply: Thanks for this suggestion. Following your advice, we fused single-nucleotide (50557 A/T/C/G) variants of the sequence flanking the putative promoter (Fig. 6E and the appended Fig. S8A) with a red fluorecent protein, then introduced the fusion construct into the non-pathogenic *M. smegmatis* using an integrative shuttle plasmid. Both fluorescent microscopy and plate reader data showed that the 50557T variant drives specific and strong expression of its downstream fluorescent protein, likely through the TACCCT -10 concensus. We believe that our new experimental validation, now presented in Supplementary Figure 8 and described in a short paragraph (lines 279–282) of the main text, further supports that the 50557C>T mutation is site- and nucleotide-specific.

6.In the heatmap of Fig. 5A, the authors compare 25 different cytological features. However, it is unclear what these 25 features represent, as only five cytological features were mentioned in earlier results.

Reply: Thanks for this comment. In this revised manuscript, we have edited the corresponding text to briefly explain these features and provided supplementary tables to denote sample-level (Table S1) and strain-level (Table S2) feature statistics and the intuitive meanings of the 25 lineage-predictive features (Table S3).

Re: mSystems00972-25R1 (**High-throughput Cytological Profiling Uncovers Genotype-Phenotype Associations in *Mycobacterium tuberculosis* Clinical Isolates**)

Dear Dr. Junhao Zhu:

Your manuscript has been accepted, and I am forwarding it to the ASM production staff for publication. Your paper will first be checked to make sure all elements meet the technical requirements. ASM staff will contact you if anything needs to be revised before copyediting and production can begin. Otherwise, you will be notified when your proofs are ready to be viewed.

Data Availability: ASM policy requires that data be available to the public upon online posting of the article, so please verify all links to sequence records, if present, and make sure that each number retrieves the full record of the data. If a new accession number is not linked or a link is broken, provide production staff with the correct URL for the record. If the accession numbers for new data are not publicly accessible before the expected online posting of the article, publication may be delayed; please contact ASM production staff immediately with the expected release date.

Publication Fees: For information on publication fees and which article types have charges, please visit our website. We have partnered with Copyright Clearance Center (CCC) to collect author charges. If fees apply to your paper, you will receive a message from no-reply@copyright.com with further instructions. For questions related to paying charges through RightsLink, please contact CCC at ASM_Support@copyright.com or toll free at +1-877-622-5543. CCC makes every attempt to respond to all emails within 24 hours.

ASM Membership: Corresponding authors may join or renew ASM membership to obtain discounts on publication fees. Need to upgrade your membership level? Please contact Customer Service at Service@asmusa.org.

Embargo Policy: A press release may be issued as soon as the manuscript is posted on the mSystems Latest Articles webpage. The corresponding author will receive an email with the subject line "ASM Journals Author Services Notification" when the article is available online.

Cover Image Submissions: If you would like to submit a potential Cover Image, please email a file and a short legend to msystems@asmusa.org. Please note that we can only consider images that (i) the authors created or own and (ii) have not been previously published. By submitting, you agree that the image can be used under the same terms as the published article. Image File requirements: TIF/EPS, 7.5 inches wide by 8.25 inches tall (at least 2,250 pixels wide by 2,475 pixels tall), minimum 300 dpi resolution (600 dpi preferred), RGB, and no figure elements, e.g., arrows or panel labels. The legend should be a short description of the image, 1-2 sentences recommended. Please download and use this interactive template in Adobe to ensure that your proposed cover image meets our size requirements (https://journals.asm.org/pb-assets/pdf-text-excel-files/ASM-Interactive-Sizing-Cover-Template-1715689791.pdf).

Thank you for submitting your paper to mSystems.

Sincerely,
Mark Mandel
Editor
mSystems
